# The role of climate in past forest loss in an ecologically important region of South Asia

**DOI:** 10.1111/gcb.16161

**Published:** 2022-03-23

**Authors:** Alice E. Haughan, Nathalie Pettorelli, Simon G. Potts, Deepa Senapathi

**Affiliations:** ^1^ School of Agriculture, Policy and Development Centre for Agri‐Environmental Research University of Reading Reading UK; ^2^ Institute of Zoology Zoological Society of London London UK

**Keywords:** climate velocity, forest loss, precipitation, regional, seasonal variation, temperature

## Abstract

Tropical forests in India have declined at an alarming rate over the past century, with extensive literature focusing on the high contributions of agricultural expansions to deforestation, while the effects of climate change have largely been overlooked. Climate change effects, such as increasing temperatures, drought and flooding, have already occurred, and are projected to worsen. Climate velocity, a metric that accounts for spatial heterogeneity in climate, can help identify contiguous areas under greater climate stress and potential climate refuges in addition to traditional temporal trends. Here, we examined the relative contribution of climate changes to forest loss within an area of India, Jammu, Kashmir, and Ladakh during the period 2001–2018, at two spatial (regional and study area) and two temporal (seasonal and annual) scales. This includes, for the first time, a characterization of climate velocity in the area. Our findings show that annual forest loss increased substantially over the 17‐year period examined (2001–2018), with the majority of forest loss occurring in the Northeast region. Decreases in temporal trends of temperature and precipitation were most associated with forest losses, but there was large spatial and seasonal variation in the relationship. In every region except the Northeast, forest losses were correlated with faster velocities of at least one climate variable but overlapping areas of high velocities were rare. Our findings indicate that climate changes have played an important role in past forest loss within the study area but likely remain secondary to other factors at present. We stress concern for climates velocities recorded reaching 97 km year^−1^, and highlight that understanding the different regional and seasonal relationships between climatic conditions and forest distributions will be key to effective protection of the area's remaining forests as climate change accelerates.

## Graphical abstract

The effects of climate change on tropical forest loss in India have largely been overlooked. Here, we examined the relative contribution of climate changes and velocities to forest loss during 2001–2018. Decreases in temperature and precipitation were most associated with forest losses but there was large spatial and seasonal variation in the relationship. Generally, forest losses were correlated with faster velocities of at least one climate variable and overlapping areas of high velocities were rare. Our findings indicate that climate changes have played an important role in India's past forest loss, but likely remain secondary to other factors at present.

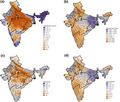

## 1 INTRODUCTION

Forests are being destroyed at an alarming rate globally (FAO & UNEP, 2020; Haddad et al., 2015; Song et al., 2018), despite their importance for human well‐being and the maintenance of planetary ecosystems. Tropical forests, home to a disproportionate amount of the world's biodiversity, are experiencing some of the largest declines (França et al., 2020; Hansen et al., 2013; IPBES, 2019; Song et al., 2018). Land use change is the leading cause of forest declines worldwide (Choe & Thorne, 2017; FAO & UNEP, 2020; Ostberg et al., 2015; WWF, 2020) with recent estimates, suggesting that only 24% of tropical forests are still intact (Lewis et al., 2015). In addition, there is an increasing concern regarding the impacts of climate change, with research suggesting that its effects could be already eclipsing those of land use change on 60% of the global land surface (Ostberg et al., 2015). Climate change effects are increasing in many areas of the world, including tropical forests (IPCC, 2019; Ostberg et al., 2015; WWF, 2020). However, there are still significant knowledge gaps around how changing climate trends over time, and the speed of climate change, may be impacting forests in terms of tree mortality, growth and productivity (Allen et al., 2010; Carnicer et al., 2011; Van Mantgem et al., 2009; Senf et al., 2018). These effects could have potential implications for biodiversity that is reliant on tropical forests, and the ecosystem services tropical forests provide in the form of carbon capture and the water cycle (Allen et al., 2010).

Impacts of climate change on forests are often largely dependent on geographical location and interactions between climate variables (Allen et al., 2010; Brito‐Morales et al., 2018; Maracchi et al., 2005) but have been shown to both positively and negatively affect forest growth, mortality, productivity and distribution, alongside impacting the capability to deal with other stressors like drought and fire (IPCC, 2019; Ovenden et al., 2021). Temperature increases are by far the most commonly studied climate driver of forest mortality (Chen et al., 2011; Heikkinen et al., 2020; Maringer et al., 2021; Seidl et al., 2017) and have been shown to directly impact forest distribution and growth (Garcia et al., 2014; Lenoir & Svenning, 2015). Changes in precipitation have also been shown to affect forest survival, most commonly precipitation decreases (Aiba & Kitayama, 2002; Bennett et al., 2015; Chen et al., 2017; Phillips et al., 2009; Taccoen et al., 2019; Zhang et al., 2017), but the relationships are often complex (Bateman et al., 2016; Seidl et al., 2017) and can be highly dependent on forest type, previous conditions and phenotypical adaptations of species (Das et al., 2013; Greenwood et al., 2017; McDowell, 2018). Tree mortality from climate change is often linked to drought‐induced hydraulic failure or carbon starvation (Allen et al., 2010; McDowell et al., 2018), but indirect effects such as increased forest susceptibility to pests and diseases (Lindner et al., 2010; Seidl et al., 2017; Stralberg et al., 2015), and human decisions surrounding land use change and resource extraction (IPBES, 2018; Liu et al., 2007) also occur. There is also evidence to suggest some climate changes that are expected to support tree growth, for example through increased CO_2_ fertilization and light exposure, can actually lead to mortality, for example, when increased growth leads to greater competition for resources (Huete et al., 2006; McDowell et al., 2018; Saleska et al., 2007). Climatic effects and contributions to tree mortality remain far less understood than effects on other forest processes, such as productivity and growth (Neumann et al., 2017; Park Williams et al., 2013).

Typically, studies assess the risk of temporal trends in climate variables, but the spatial heterogeneity of the landscape can also be important. Climate velocity (Loarie et al., 2009), a metric that encompasses the spatial heterogeneity in climate in the surrounding area, theorizes that areas where climate is changing quickly and similar climates are further away, will be at greater risk to climate change (García Molinos et al., 2019; Garcia et al., 2014; Hamann et al., 2015; Loarie et al., 2009). The metric provides an additional dimension to climate risk, and subsequently high velocities have been linked to reductions and redistributions in small‐ranged species (Sandel et al., 2011), marine taxa (García Molinos et al., 2016), birds (Bateman et al., 2016) and trees (Bateman et al., 2016; Liang et al., 2018; Nadeau & Fuller, 2015; Sandel et al., 2011), and areas of low velocities have been hailed potential climate refuges (Brito‐Morales et al., 2018; Heikkinen et al., 2020). Climate velocity estimates may be an important component for identifying areas most at risk to the effects of climate change, providing a dimension that temporal trends cannot (Garcia et al., 2014; Heikkinen et al., 2020; Loarie et al., 2009).

Currently, there is a strong bias in the literature on climate change effects on forest systems towards northern temperate regions, particularly for velocity studies, and tropical forests have been less studied in comparison (Brito‐Morales et al., 2018; França et al., 2020; Lenoir & Svenning, 2015; Seidl et al., 2017). Drawing conclusions about the effect of climate change in tropical regions is often more complex than the temperate counterparts, in part due to a large variety of forest types, adaptations and microclimates, and a lower availability to high‐quality data (McDowell, 2018). In the past, many studies have focused on Amazonia (Giardina et al., 2018; Huete et al., 2006; Nepstad et al., 2007; Saleska et al., 2007), where deforestation rates are the highest. These studies have found that increasing temperatures and decreasing precipitation resulted in tropical trees being more susceptible to mortality, either through heat stress and drought (Giardina et al., 2018; Nepstad, 2007; Phillips et al., 2009), or increased fire risk (Brando et al., 2014). However, evidence suggests that responses across tropical regions may be highly diverse and can be dependent on tree characteristics such as growth rate, deciduousness and root depth (Asner et al., 2010; McDowell, 2018; Wagner et al., 2014). Though the majority of tropical studies assessing climate change effects on forests remain in Amazonia, there are several studies that have focused on other parts of the tropics. For example, studies have shown increased forest mortality following extreme drought events in Malaysian Borneo (Nakagawa et al., 2001), Indonesian Borneo (Van Nieuwstadt & Sheil, 2004) and Northwest India (Khan et al., 1994). Studies from Africa have also shown changes in the distribution of forests as a result of long‐term changes in precipitation and temperature regimes (Biasutti & Giannini, 2006; Foden et al., 2007; Gonzalez, 2001).

India, where the majority of the study area is located, is in the top 10 countries in the world for forest cover (FAO & UNEP, 2020). Forests, primarily tropical and sub‐tropical, cover 20% of the country's land mass (Ravindranath et al., 2005). It is one of the most biodiverse countries in the world, representing 11% of the world's flora and encompassing four biodiversity hotspots (Chitale et al., 2014; NWAP, 2017). The country has experienced large‐scale forest loss for decades, which has been extensively studied, with land use changes largely cited as the major cause of forest declines (Jha et al., 2000; Lele & Joshi, 2009; Reddy et al., 2013; Roy et al., 2013). Increased demand for crop productions, commercial livestock rearing, timber extraction, rapidly increasing populations and an emerging economy are all known to be putting high pressure on forests, alongside cultural practices of shifting cultivation (Lele & Joshi, 2009; Wani et al., 2012). Large proportions of the population directly rely on forests for their survival and livelihoods, and in particular, fuelwood and fodder collection are major sources of domestic energy and income for tens of thousands of villages (Roy et al., 2013; Sharma et al., 2015). Whereas the effect of land use change is well documented (Davidar et al., 2010; Gupta, 2007; Lele & Joshi, 2009; Roy et al., 2013), there has been little focus on the role of climate change to past forest loss. Ascertaining climate's role in the country's past forest loss could help predict the future stability of forests in the face of increasing change, as well as aiding effective management strategies for current forest conservation. Due to the unique variation in climate driven by two monsoon systems (Krishnan et al., 2020), the study area is likely to experience a range of different climate changes and is therefore an ideal location to study the effects of climate change, including velocity, on tropical forest systems.

Climate change in India has been evident for many years and numerous studies have described a consistent pattern of warming (Dash et al., 2011; Mishra, 2019; Rao et al., 2016; Ravindranath et al., 2011; Rupa Kumar et al., 2006), more frequent high‐intensity rain events, higher maximum temperatures (Krishnan et al., 2020), warmer winters and a lower confidence in the timing of the monsoon which is critical for India's agricultural‐driven economy (Dash et al., 2011; Ravindranath et al., 2011). Research that focuses on the relationship between climate change and forest loss in the study area has almost always analysed the potential threats of *future* climate change on forests through global vegetation models (Brown & Pearce, 1994; Chaturvedi et al., 2011; Gopalakrishnan et al., 2011; Kumar et al., 2018; Ravindranath et al., 2005; Sharma et al., 2017; Upgupta et al., 2015), but none so far have considered velocity. Existing studies have predicted climate change to have strong influences on forest cover, consistently predicting a shift to wetter forest types and a loss of drier forest types in response to a generally warmer and wetter climate in the future, noting precipitation thresholds to be particularly important (Chaturvedi et al., 2011; Gopalakrishnan et al., 2011; Ravindranath & Sukumar, 1998; Ravindranath et al., 2005). Some regions are predicted to gain forests, while others, to lose forest (Chaturvedi et al., 2011; Ravindranath et al., 2005). Areas of highest vulnerability are those with projected increases in temperature but decreases in precipitation (Chaturvedi et al., 2011). Past research has generally predicted the Himalayan forests, northern Western Ghats and North‐western regions of India to be most at risk to climate change effects due to a combination of forest intactness, forest type and climate change exposure (Chaturvedi et al., 2011; Gopalakrishnan et al., 2011; Upgupta et al., 2015). Whereas forests in the north‐eastern region and southern Western Ghats are expected to be less vulnerable due to being predominantly composed of tropical moist forests which are likely to expand in range, alongside higher levels of intactness and species richness (Chaturvedi et al., 2011; Gopalakrishnan et al., 2011; Ravindranath et al., 2005).

While these projections provide useful foresight into potential at‐risk areas, there is a clear gap in our understanding of the distribution of climatic effects in areas of high forest loss in the past which could help inform future predictions. Additionally, mapping and analysing the distribution in climate velocity in an area could be crucial for conservation strategies to support in‐situ adaptation, by limiting other stressors, considering potential strategies for relocating or aiding limited dispersal to less affected areas.

This study aims to characterize the relationship between climate change and past forest loss within the study area and explores the relative importance of drivers other than the well‐documented effects of land use change. It aims to map and analyse climate velocities for the first time in India, Jammu, Kashmir and Ladakh and critically assess the usefulness of this metric in providing additional understanding of risks to forests. Given current evidence, we expect climate changes, such as declining precipitation and temperature increases, to be correlated with areas of high forest loss. However, we expect considerable seasonal and regional variation due to the diversity in climate and geography across the study area, which we account for in our methodology. Though previous analyses assessing the effect of climate velocity on ecosystems have been largely confined to higher latitude studies (Dial et al. 2016; Dobrowski et al. 2012; Dobrowski & Parks, 2016; Kosanic et al. 2019), evidence from these and coarser‐scale global analyses (Burrows et al. 2014; Loarie et al. 2009) lead us to suspect that climate velocity may have a significant effect on forest distributions and survival. We expect that forest loss will be greater in areas of higher climate velocity where forests are more exposed to faster changes in climate or where high velocities of multiple variables overlaps.

The key questions addressed in this manuscript are:

1. Is there a relationship between climate change and past forest loss?
2. Are there seasonal and regional variations in the climate–forest loss relationship in the study area?
3. Are forests in the study area exposed to high and/or overlapping climate velocities and is forest loss greater in these areas?
4. Can climate velocity provide additional understanding of forests’ risk to climate change?

## 2 METHODS

### 2.1 Forest loss

Records of annual forest loss were obtained from the Hansen Global Forest Change v1.6 dataset (GFC) (Hansen et al., 2013) for the period 2001–2018 at a spatial resolution of ~30 m, within the Google Earth Engine interface (Gorelick, et al., 2017). The GFC data take the form of a binary record of loss (1) or no loss (0) for each pixel in the area of interest and recording all trees above 5 m in height. District level totals of forest loss (km²) were generated for all 577 districts encompassing parts of India, Jammu, Kashmir and Ladakh and subsequently analysed in r (version 4.03; R Core Team, 2017). Districts are a political boundary and districts sit within a state boundary. The State level is where most political decisions are made. The 577 districts included in the analysis accounted for an area of 3,455,785 km^2^. Any districts with less than a total of 0.1 km² forest cover were excluded to avoid any noise in the Hansen GFC data. This resulted in a total of 13 districts (of 577) being excluded from the analyses, predominantly from the arid and xeric shrubland regions of the Northwest (Table S1). These 13 excluded districts covered a land area that accounted 5.73% of the total land area in the study and 0.0001% of the area's forest cover. In addition to those removed for low levels of forest cover, island union territories were excluded due to potential differences between island and land mass effects of climate in addition to concern over the accuracy of the datasets used on small island states.

### 2.2 Climate data

Global raster datasets of total monthly precipitation (mm) and monthly mean temperature (^o^C) from the Climate Research Unit (CRU TS v. 4.03) were obtained at 0.5 × 0.5 degree resolution (~112 km^2^), covering the years 2001–2018. The selected period was chosen to align with the availability of GFC data. Climate datasets were averaged to create a data point for each district. Regional datasets were also created by compiling districts belonging to each of the six monsoon regions outlined by the Indian Institute of Tropical Meteorology (www.tropmet.res.in); Northeast (NE), Northwest (NW), Central Northwest (CNE), West Central (WC), Peninsular (PEN) and Hilly region, composed of the East Hilly Region (EHR) and the West Hilly Region (WHR) (Figure S1). The monthly data were aggregated to create a dataset of total annual precipitation by calculating, for each raster cell in each year, the sum of the monthly values. The monthly data were also aggregated to create a dataset of mean temperature for each year averaging a cells value across all months of the year.

For the seasonal analysis, data were collated from the monthly climate rasters and averages of mean temperature and total precipitation calculated for each season at both the study area and regional spatial scales. The seasons are those used by the Indian Meteorological Department (http://www.imdpune.gov.in/Weather/Reports/glossary.pdf) and most commonly found in the literature for national‐scale studies of India, where the majority of the study area resides. These were monsoon (June–September), post‐monsoon (October–December), winter (January–February) and pre‐monsoon (March–May). It is important to note that, despite these being the standard national seasons, the climate of each season varies considerably by region (Figures S2 and S3).

### 2.3 Calculating climate velocity

Gradient‐based climate velocity was calculated in r using the gvocc package and the integrated functions; SpatGrad and TempTrend following the methodology for local climate velocity outlined in García Molinos et al. (2019) and based off the original calculation by Loarie et al. (2009). The TempTrend function calculates the temporal trend by performing linear regressions of the variable against time for each individual cell. This was calculated for both the annual and seasonal averages separately. The temporal trends were used in the climate velocity metric but also as a separate variable in the models. The SpatGrad function calculates spatial gradients for each cell by determining the magnitude of the differences in the climate variable over its neighbouring (3 × 3) cells. To avoid the potential of infinite velocities caused by spatial gradients of zero (Hamann et al., 2015; Loarie et al., 2009), a value of 0.1 was added to all the data points. Climate velocity was then calculated by dividing the temporal trend by the spatial gradient. An average climate velocity for each variable was calculated per district by taking the mean magnitude from all the cells present in a district's boundary using the zonal statistics function in QGIS v3.8.2 (QGIS.org, 2019). Each district was an individual data point used in the models.

It is important to note that climate velocity can be both negative and positive—the direction of the effect is taken from the temporal trend, and it is the magnitude that relates to the velocity. So, a large negative precipitation velocity indicates a faster reduction in precipitation over time, and a large positive precipitation velocity indicates a faster increase in precipitation. Smaller velocities indicate slower changes. Therefore, positive relationships between forest loss and climate velocity could equally represent greater forest loss at faster positive velocities or slower negative velocities, whereas negative relationships represent greater forest loss at faster negative velocities or slower positive velocities.

The areas of high climate velocities were also defined. These were characterized as the fastest 10% of positive and negative values, separately, for each season. These were used to assess the overlap between high temperature and precipitation velocities and map areas where forests may be under additional pressure from experiencing high velocities of both climate variables.

### 2.4 Population density as a proxy for human pressures

Human pressures, particularly land use changes, are regularly cited as a primary cause of forest loss in the study area. To account for the effects of these, a proxy of population density was included as an explanatory variable (Cimatti et al., 2021; Kok, 2004; Milanesi et al., 2017). Population density has been shown to have a large effect on land use changes in the study area in the past, particularly relating to forest cover, agriculture and urban areas (Kale et al., 2016; Palchoudhuri et al., 2015). Data on population density (people per km²) for the years 2000 and 2020 were obtained from SEDAC’s GPWv4.11 dataset at a spatial resolution of 30 arc‐seconds (~1 km^2^ at the equator). Population density change over the 20‐year period was calculated on a cell‐by‐cell basis by subtracting the final year's values from the first year. Cells with positive values represented an increase in population density over time and cells with a negative value, a decrease. Mean values of population density change were calculated for each district from this cell‐level data. The data were only available in 5‐year increments; thus, the years 2000 and 2020 were selected to match the forest loss data as closely as possible (Figure S4). Population density change was included as a fixed effect in the model.

### 2.5 Modelling the impact of climate change on forest loss

Linear mixed‐effects models were developed using the nlme package in r to assess the relationship of the climate variables on forest loss at both the study area and regional levels. First, a null model comprising of the response variable (forest loss per district in km^2^) and a random effect of the State (political boundary) was created as a basis for model generation. The state that the forest belonged to was considered to affect the level of forest loss due to the individual forest policies between states; subsequently, districts in the same state are likely to be more similar.

Four model structures were created in total. Of these four, two included temperature and precipitation velocity as the explanatory variable, with one using annual and the other using seasonal data. The second two models included the temporal trends of temperature and precipitation as the explanatory variables, again one using annual data and the other seasonal data. The models were applied to data at the study area scale and then to data for each of the six homogeneous monsoon regions to provide analysis at two spatial scales.

All models included population density change as an explanatory variable and state as a random effect. Subsequently, the seasonal models (temporal trend and velocity) included nine fixed effects: the temperature and precipitation metric for each of the four seasons, and population density change (Tables 1, 2, 3). The annual models included three fixed effects: the average annual temperature metric, the average annual precipitation metric and population density change.

**TABLE 1:** Study area‐scale seasonal models of the effects of climate velocity and temporal trends on forest loss, accounting for population density. The response variable tested in each model was forest loss (km^2^). The explanatory variables were the fixed effects of the eight seasonal climate variables, population density change between 2000 and 2020 (people per km^2^) and a random effect of State (political boundary). The table highlights a list of the variables included in each model under the ‘All fixed effects variables tested’ column. The ‘Significant fixed effect variables’ column shows those that had a significant effect on forest loss

Model	All fixed effects variables tested	Significant fixed effect variables	Estimate	*t*‐value	*p*‐value
Seasonal velocity	Pre‐monsoon precipitation (mm km^−1^), monsoon precipitation (mm km^−1^), post‐monsoon precipitation (mm km^−1^), winter precipitation (mm km^−1^), pre‐monsoon temperature (°C km^−1^), monsoon temperature (°C km^−1^), post‐monsoon temperature (°C km^−1^), winter temperature (°C km^−1^), population density change (people per km^2^)	Pre‐monsoon precipitation	0.088	2.128	.033
Marginal *R* ^2^ = .059 Conditional *R* ^2^ = .621		Winter precipitation	−0.121	−2.225	.026
		Monsoon temperature	−0.335	−4.007	<.001
		Winter temperature	0.384	2.363	.018
Seasonal temporal trends	Pre‐monsoon precipitation (mm year^−1^), monsoon precipitation (mm year^−1^), post‐monsoon precipitation (mm year^−1^), winter precipitation (mm year^−1^), pre‐monsoon temperature (°C year^−1^), monsoon temperature (°C year^−1^), post‐monsoon temperature (°C year^−1^), winter temperature (°C year^−1^), population density change (people per km^2^)	Pre‐monsoon precipitation	−0.251	−3.544	<.001
		Pre‐monsoon temperature	−0.341	−2.395	.016
Marginal *R* ^2^ = .122 Conditional *R* ^2^ = .523					
		Monsoon temperature	−0.383	−3.863	<.001
		Winter temperature	0.507	3.027	.002
		Population density change	−0.083	−2.381	.017

**TABLE 2:** Regional‐scale seasonal models of the effects of climate velocities on regional forest loss accounting for population density. The response variable tested in each model was forest loss (km^2^). The explanatory variables were the fixed effects of the eight seasonal climate velocities and population density change between 2000 and 2020 (people per km^2^) and a random effect of State (political boundary). The table highlights a list of the variables included in each model under the ‘All fixed effects variables tested’ column. The ‘Significant fixed effect variables’ column shows those that had a significant effect on forest loss

Model	All fixed effects variables tested	Significant fixed effect variables	Estimate	*t*‐value	*p*‐value
NE	Pre‐monsoon precipitation (mm km^−1^), monsoon precipitation (mm km^−1^), post‐monsoon precipitation (mm km^−1^), winter precipitation (mm km^−1^), pre‐monsoon temperature (°C km^−1^), monsoon temperature (°C km^−1^), post‐monsoon temperature (°C km^−1^), winter temperature (°C km^−1^), population density change (people per km^2^)	No significant variables	NA	NA	NA
Marginal *R* ^2^ = .268 Conditional *R* ^2^ = .319					
CNE	Pre‐monsoon precipitation (mm km^−1^), monsoon precipitation (mm km^−1^), post‐monsoon precipitation (mm km^−1^), winter precipitation (mm km^−1^), pre‐monsoon temperature (°C km^−1^), monsoon temperature (°C km^−1^), post‐monsoon temperature (°C km^−1^), winter temperature (°C km^−1^), population density change (people per km^2^)	Pre‐monsoon precipitation	0.831	5.438	<.001
Marginal *R* ^2^ = .340 Conditional R^2^ = .343					
NW	Pre‐monsoon precipitation (mm km^−1^), monsoon precipitation (mm km^−1^), post‐monsoon precipitation (mm km^−1^), winter precipitation (mm km^−1^), pre‐monsoon temperature (°C km^−1^), monsoon temperature (°C km^−1^), post‐monsoon temperature (°C km^−1^), winter temperature (°C km^−1^), population density change (people per km^2^)	Monsoon precipitation	0.295	2.414	.018
		Monsoon temperature	−0.634	−3.267	.001
Marginal *R* ^2^ = .339 Conditional *R* ^2^ = .672					
		Post‐monsoon temperature	0.674	3.258	.001
WC	Pre‐monsoon precipitation (mm km^−1^), monsoon precipitation (mm km^−1^), post‐monsoon precipitation (mm km^−1^), winter precipitation (mm km^−1^), pre‐monsoon temperature (°C km^−1^), monsoon temperature (°C km^−1^), post‐monsoon temperature (°C km^−1^), winter temperature (°C km^−1^), population density change (people per km^2^)	Post‐monsoon temperature	0.670	2.279	.024
Marginal *R* ^2^ = .100 Conditional *R* ^2^ = .154					
PEN	Pre‐monsoon precipitation (mm km^−1^), monsoon precipitation (mm km^−1^), post‐monsoon precipitation (mm km^−1^), winter precipitation (mm km^−1^), pre‐monsoon temperature (°C km^−1^), monsoon temperature (°C km^−1^), post‐monsoon temperature (°C km^−1^), winter temperature (°C km^−1^), population density change (people per km^2^)	Post‐monsoon temperature	−0.463	−3.799	<.001
Marginal *R* ^2^ = .461 Conditional *R* ^2^ = .522					
Hilly	Pre‐monsoon precipitation (mm km^−1^), monsoon precipitation (mm km^−1^), post‐monsoon precipitation (mm km^−1^), winter precipitation (mm km^−1^), pre‐monsoon temperature (°C km^−1^), monsoon temperature (°C km^−1^), post‐monsoon temperature (°C km^−1^), winter temperature (°C km^−1^), population density change (people per km^2^)	Post‐monsoon precipitation	−0.589	−3.087	.003
		Winter precipitation	0.571	3.565	.001
Marginal *R* ^2^ = .612 Conditional *R* ^2^ = .632					
		Monsoon temperature	−0.594	−2.553	.014

**TABLE 3:** Regional‐scale seasonal models of the effects of climatic temporal trends on regional forest loss. The response variable tested in each model was forest loss (km^2^). The response variable tested in each model was forest loss (km^2^). The explanatory variables were the fixed effects of the eight seasonal climate temporal trends and population density change between 2000 and 2020 (people per km^2^) and a random effect of State (political boundary). The table highlights a list of the variables included in each model under the ‘All fixed effects variables tested’ column. The ‘Significant fixed effect variables’ column shows those that had a significant effect on forest loss

Model	All fixed effects variables tested	Significant fixed effect variables	Estimate	*t*‐value	*p*‐value
NE	Pre‐monsoon precipitation (mm year^−1^), monsoon precipitation (mm year^−1^), post‐monsoon precipitation (mm year^−1^), winter precipitation (mm year^−1^), pre‐monsoon temperature (°C year^−1^), monsoon temperature (°C year^−1^), post‐monsoon temperature (°C year^−1^), winter temperature (°C year^−1^), population density change (people per km^2^)	Monsoon temperature	−0.626	−2.353	.021
Marginal *R* ^2^ = .313 Conditional *R* ^2^ = .339					
		Population density change	−0.242	−2.076	.041
CNE	Pre‐monsoon precipitation (mm year^−1^), monsoon precipitation (mm year^−1^), post‐monsoon precipitation (mm year^−1^), winter precipitation (mm year^−1^), pre‐monsoon temperature (°C year^−1^), monsoon temperature (°C year^−1^), post‐monsoon temperature (°C year^−1^), winter temperature (°C year^−1^), population density change (people per km^2^)	No significant variables	NA	NA	NA
Marginal *R* ^2^ = .152 Conditional *R* ^2^ = .153					
NW	Pre‐monsoon precipitation (mm year^−1^), monsoon precipitation (mm year^−1^), post‐monsoon precipitation (mm year^−1^), winter precipitation (mm year^−1^), pre‐monsoon temperature (°C year^−1^), monsoon temperature (°C year^−1^), post‐monsoon temperature (°C year^−1^), winter temperature (°C year^−1^), population density change (people per km^2^)	Pre‐monsoon precipitation	0.588	2.706	.008
		Monsoon precipitation	0.275	2.028	.046
		Winter precipitation	−1.206	−3.153	.002
Marginal *R* ^2^ = .504 Conditional *R* ^2^ = .564					
		Pre‐monsoon temperature	0.533	2.245	.027
		Winter temperature	−0.639	−2.517	.014
WC	Pre‐monsoon precipitation (mm year^−1^), monsoon precipitation (mm year^−1^), post‐monsoon precipitation (mm year^−1^), winter precipitation (mm year^−1^), pre‐monsoon temperature (°C year^−1^), monsoon temperature (°C year^−1^), post‐monsoon temperature (°C year^−1^), winter temperature (°C year^−1^), population density change (people per km^2^)	Post‐monsoon precipitation	−0.680	−2.96	.003
Marginal *R* ^2^ = .260 Conditional *R* ^2^ = .278					
PEN	Pre‐monsoon precipitation (mm year^−1^), monsoon precipitation (mm year^−1^), post‐monsoon precipitation (mm year^−1^), winter precipitation (mm year^−1^), pre‐monsoon temperature (°C year^−1^), monsoon temperature (°C year^−1^), post‐monsoon temperature (°C year^−1^), winter temperature (°C year^−1^), population density change (people per km^2^)	Monsoon temperature	−0.542	−2.222	.029
Marginal *R* ^2^ = .352 Conditional *R* ^2^ = .427					
		Post‐monsoon temperature	−0.459	−2.715	.008
Hilly	Pre‐monsoon precipitation (mm year^−1^), monsoon precipitation (mm year^−1^), post‐monsoon precipitation (mm year^−1^), winter precipitation (mm year^−1^), pre‐monsoon temperature (°C year^−1^), monsoon temperature (°C year^−1^), post‐monsoon temperature (°C year^−1^), winter temperature (°C year^−1^), population density change (people per km^2^)	No significant variables	NA	NA	NA
Marginal *R* ^2^ = .609 Conditional *R* ^2^ = .622					

In all models, the explanatory variables were standardized to account for the large variation in scale and a Gaussian spatial autocorrelation structure was used to account for spatial autocorrelation detected in the data (Moran's I *p *< .001) which was shown to adequately account for the autocorrelation with a further Moran's I test on the model residuals (Moran's I *p *> .05). Correlations between all variables were checked before inclusion in the models using Pearson's correlation coefficient. All combinations of variables had a correlation of <0.7. Marginal and conditional *R* ^2^ for each model was calculated using the methodology from Nakagawa and Schielzeth (2013) and included in the model results.

## 3 RESULTS

### 3.1 Trends in forest loss

**FIGURE 1:**
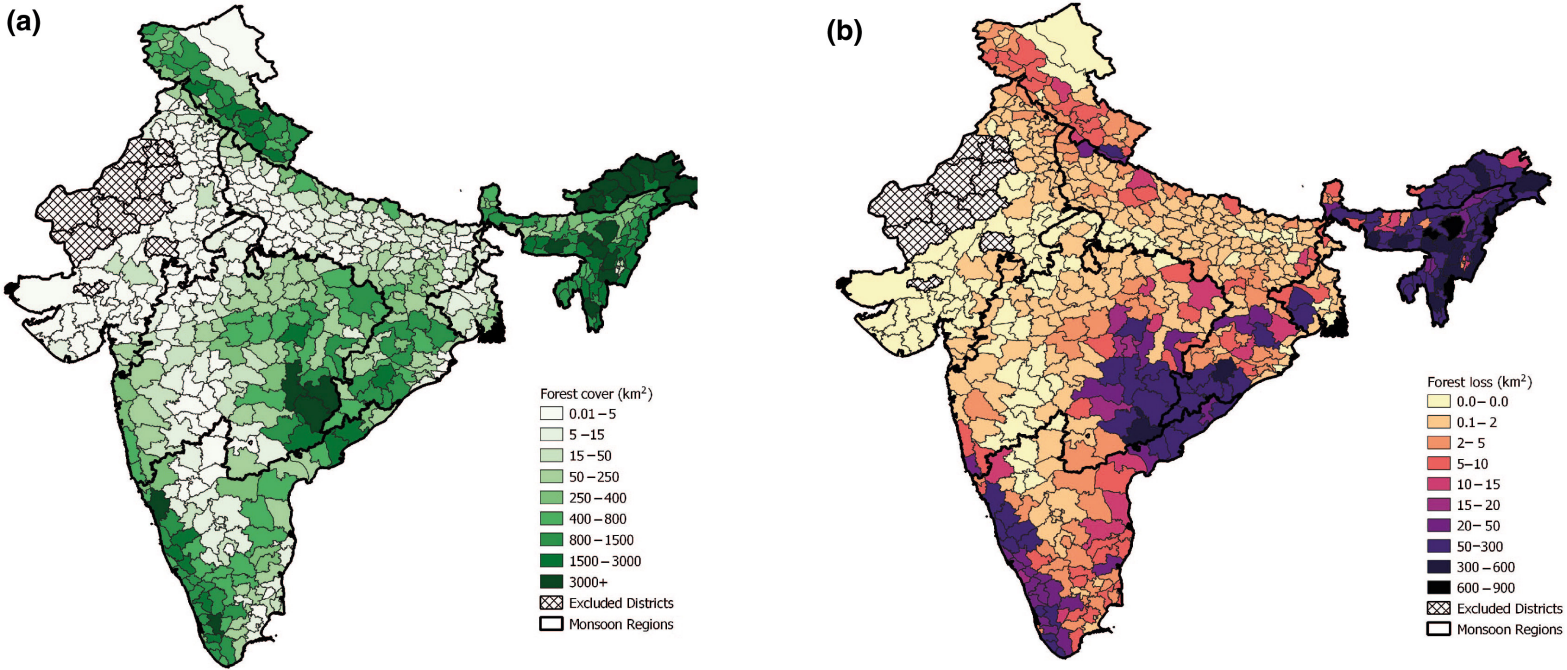
(a) Forest cover in km^2^ of each district in the study area (comprising parts of India, Jammu, Kashmir, and Ladakh) in the year 2000. (b) The total forest lost in each district between the years 2001–2018 in km^2^. Much of the forest cover is located in the Northeast and along the east and southwestern coasts. Total forest loss is greatest in the Northeast, central west coast and southwestern areas, where forest cover is also high

Forest loss increased substantially during the study period (2001–2018), escalating from annual losses of 647 km^2^ to a peak of 2,503 km^2^ lost in 2017, shortly followed by a slight decline to ~1,900 km^2^ in 2018 (Figure S5). Over the course of the 2001–2018 study period, a total of 20,472 km^2^ of forest was lost, accounting for 7.34% of the forest cover in 2001. The Northeast region contributed a significant proportion of the loss, in the last 5 years of the study losses here were over four times that of the other regions (Figure 1 and Figure S6). Three key areas of high forest losses were identified, these were as follows: (1) the combined regions of the NE and EHR, (2) the nexus of the CNE, WC and PEN regions and (3) a few districts in the northern Western Ghats (PEN region). All experienced losses >20 km^2^ over the time period (Figure S8).

### 3.2 Trends in climatic variables

#### 3.2.1 Precipitation

**FIGURE 2:**
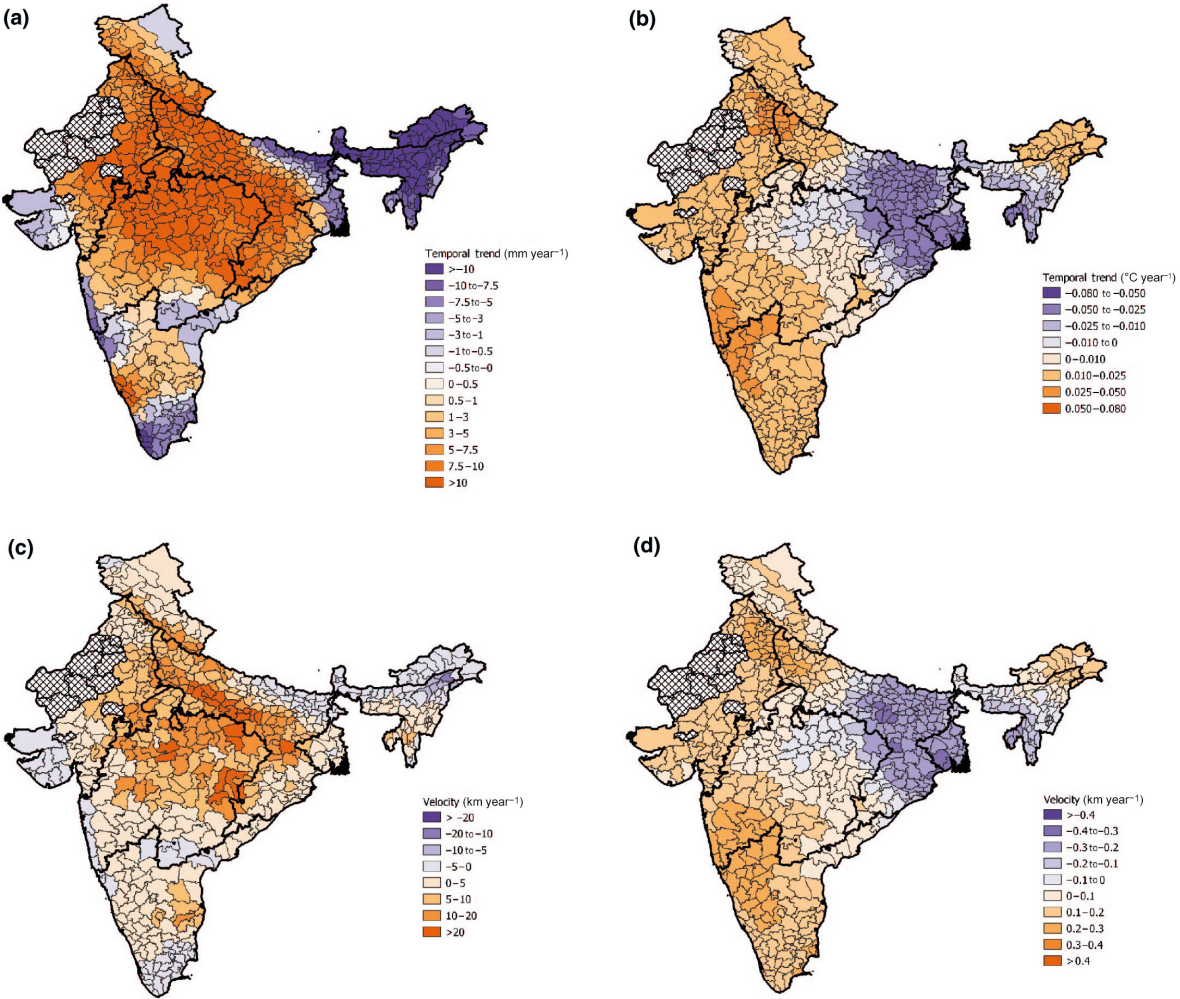
(a) Annual‐based precipitation and (b) temperature temporal trends, and (c) precipitation and (d) temperature velocities (km year^−1^) across the districts of the study area, with the outlines of the monsoon regions. Hatched districts are those that have been excluded from the study

**FIGURE 3:**
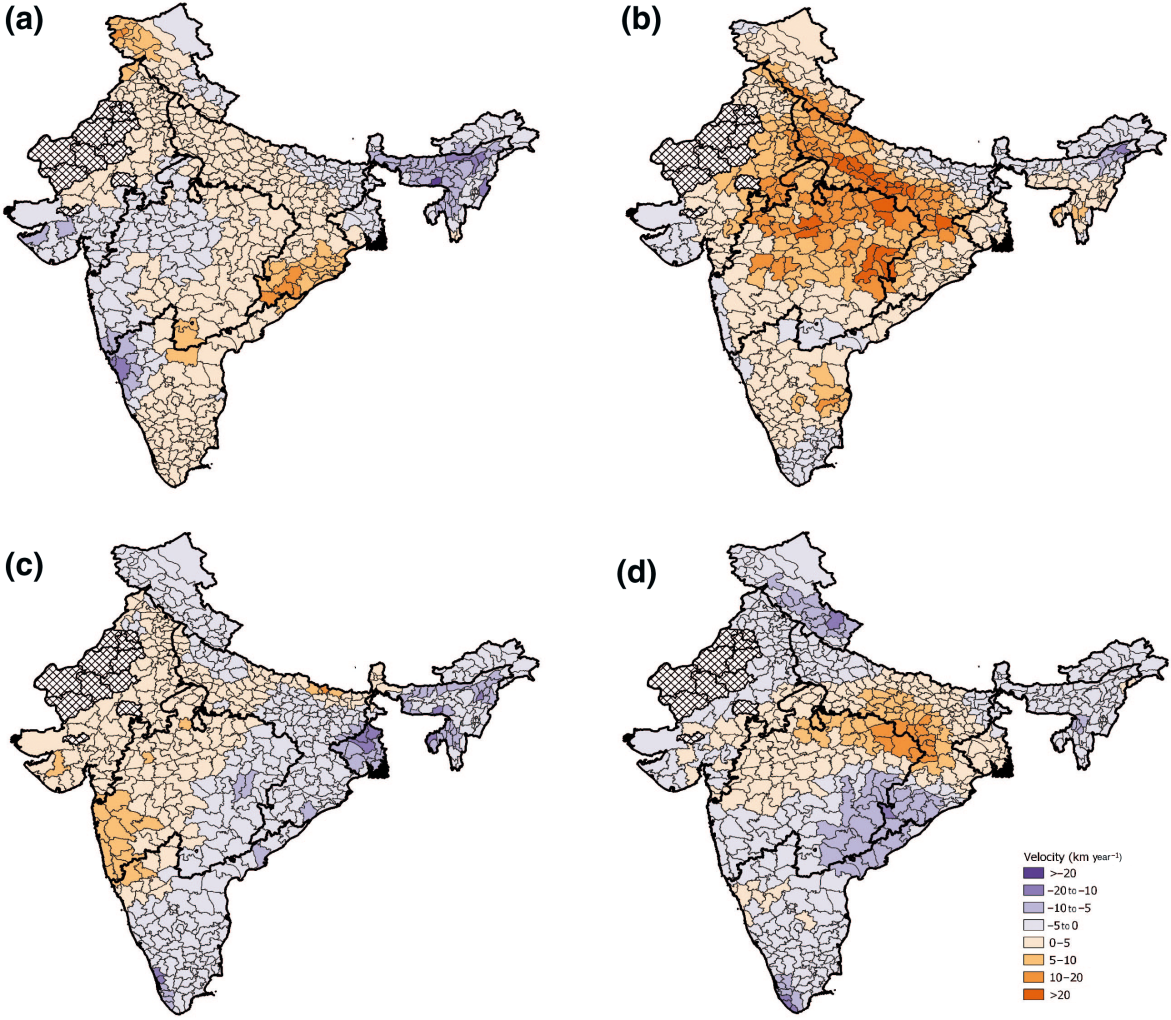
Seasonal precipitation velocities in km year^−1^ of each district for the time period 2001–2018. In a clockwise direction, the seasons depicted are as follows: pre‐monsoon (a), monsoon (b), post‐monsoon (c) and winter (d). The black outlines show the borders of the monsoon regions. Hatched districts are those that have been excluded from the study

Annual‐based temporal trends showed increases in precipitation of ~5–10 mm year^−1^ for much of the study area, with some notable exceptions in districts in the northeast and southern areas (Figure 2). Annual trends were largely driven by substantial increases recorded in the monsoon season, and the remaining three seasons showed mean decreases in precipitation (Figure S10). The same trend was found for velocities, where at times monsoon velocities reached twice the speed of other seasons (Figure 3), while the other seasons were, on the whole, getting drier but at a slower rate. Annual velocities ranged from −13 to 34 km year^−1^ (Table S2), with the fastest velocities found in districts bordering the WC and CNE regions. Seasonal velocities ranged from −97 to 41 km year^−1^ with the fastest velocities were found in the pre‐monsoon (−) and monsoon seasons (+). The most extreme velocity recorded in the study of −97.59 km year^−1^ was located in the East Khasi Hills district of the NE region during the pre‐monsoon season. Patterns of seasonal precipitation velocity were generally complex with many regions experiencing both positive and negative precipitation velocities at different points in the year (Figure 3).

#### 3.2.2 Temperature

**FIGURE 4:**
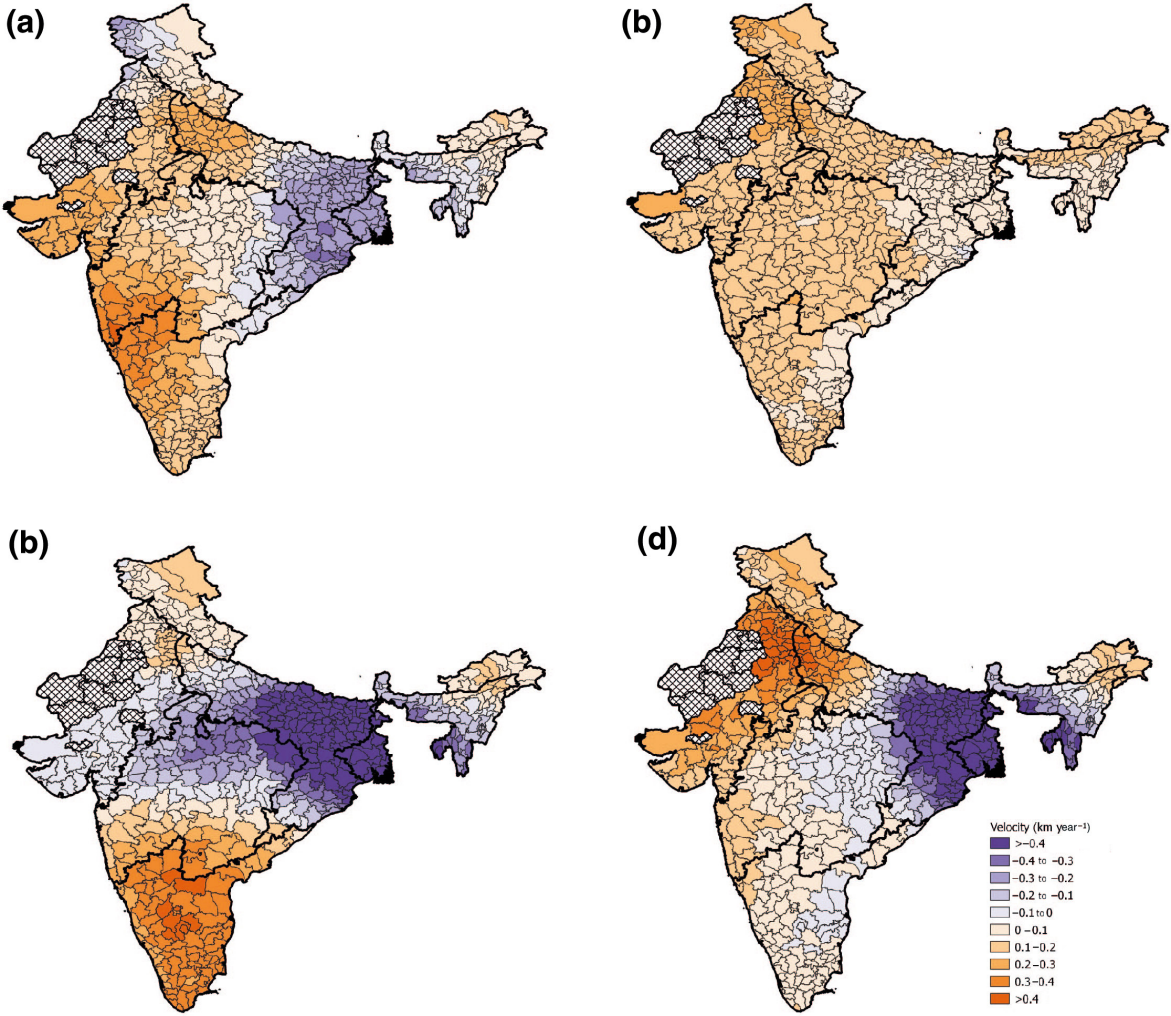
Seasonal temperature velocities in km year^−1^ of each district for the time period 2001–2018. In a clockwise direction, the seasons depicted are as follows: pre‐monsoon (a), monsoon (b), post‐monsoon (c) and winter (d). The black outlines show the borders of the monsoon regions. Hatched districts are those that have been excluded from the study

Based on annual temperature temporal trends, the majority of the study area warmed at a rate around 0.025–0.050°C year^−1^, with notable exceptions of some CNE and NE districts where temperature was cooling (Figure 2). Seasonal analyses showed the fastest warming to be in the winter season where some districts exceeded increases of 0.051°C year^−1^. There were no occurrences of temperature reductions in the monsoon season. Seasonal variation was greater for precipitation than temperature with many regions experiencing the same temperature trends year‐round. Annual‐based temperature velocities ranged between −0.321 and 0.298 km year^−1^ (Table S2) and followed a similar patterning to the temporal trends. The highest positive velocities rotated around the study area throughout the year resulting in high but seasonal exposure to fast positive velocities in much of the North, West and South (Figure 4). The fastest negative velocities of −0.4 km year^−1^ were located in the CNE and NE regions. Temperature velocities were much slower than those recorded for precipitation.

### 3.3 The influence of spatial gradients on climatic trends

Spatial gradients differed between temperature and precipitation variables as well as between seasons, leading to a variety of differences in temporal trends and velocities between the two variables (Table S3). The patterns of velocities often matched those of their temporal trend counterparts, but velocity magnitudes were found to be greatly affected by spatial gradients. In some cases, trends were reversed due to the influence of spatial gradients. For example, a dampening of the negative pre‐monsoon precipitation temporal trend in the NE due to a high spatial gradient alongside an exacerbation of a positive temporal trend in the southern CNE region led to a different relationship between pre‐monsoon precipitation and forest loss in the temporal trend and velocity models. Effects of spatial gradients were more evident for precipitation than temperature which had lower spatial heterogeneity in climate.

### 3.4 Statistical models

At the study area scale, there was no effect of annual‐based climate change on forest loss. However, there were significant effects of seasonal climate changes on forest loss. Velocities of monsoon temperature and temporal trends of monsoon temperature, pre‐monsoon temperature and pre‐monsoon precipitation showed significant effects on forest loss with a negative effect direction. While velocities of winter temperatures, pre‐monsoon precipitation and winter precipitation, and temporal trends of winter temperatures showed significant effects on forest loss with a positive effect direction (Table 1).

In the regional models, climate was found to significantly affect forest loss in every region. Some regions were more affected than others, for example, the Northwest region (Tables 2 and 3), and each region had different compositions of climate trends that affected forest loss. The correlation between declines or lower values of monsoon temperatures and increases in forest losses was consistent across the models but other variables showed trends of both negative and positive effect directions depending on season and location.

### 3.5 The extent of overlapping climate velocities

**FIGURE 5:**
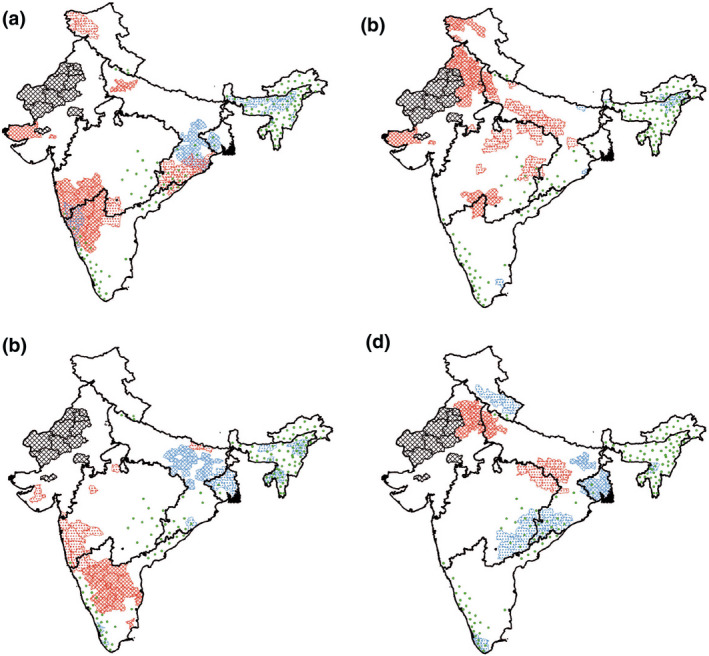
Overlaps between districts containing the 10% fastest climate velocities and the highest forest losses (>20 km^2^). In a clockwise direction, the seasons depicted are as follows: pre‐monsoon (a), monsoon (b), post‐monsoon (c) and winter (d). Positive velocities are depicted in red and negative velocities depicted in blue. Hashed districts represent temperature velocities and dots represent precipitation. Districts with the highest levels of forest loss over the time period are shown in green. The black outlines show the borders of the monsoon regions. Hatched districts are those that have been excluded from the study

In addition to the velocities of temperature and precipitation being highly variable across the study area and between seasons, they had very different spatial configurations. Overlaps between high (top 10% of values) velocities of precipitation and temperature were rare with only two instances occurring within high forest loss areas (Figure 5). The first, and largest, instance was in the Northern Western Ghats which experienced both high velocities of precipitation declines, and temperature increases during the pre‐monsoon season. The second instance occurred in the NE region where high velocities of declining precipitation overlapped with high velocities of declining temperature during the post‐monsoon season. In both the annual and winter data, no overlaps of high velocity areas were recorded. Though overlaps were rare, many areas of high forest loss experience singular high velocities over the period.

### 3.6 Population density change

Population density changes ranged from −45 to 4,000 people per km^2^, with an average increase of 200 people per km^2^. The highest increases were mainly found in the Central North‐Eastern region. Only 11 districts in the study area experienced a reduction in population density during the period (Figure S4).

Population density change did not have a significant effect on forest loss in the annual‐based study area‐scale models, but there was a negative correlation between density change and forest loss in the seasonal models. In the regional models, there was also a significant negative correlation between population density change and forest loss in the Northeast region.

## 4 DISCUSSION

This study indicates that climate change has played a significant role in forest loss within the study area, a contribution that has previously been overlooked. This study highlights the complexities of climate change effects on forests, the emerging climatic trends that may cause risks to forests in the future and analyses the relevance of velocity metrics in tropical forest systems. Here, the findings are discussed in relation to the research questions.

### 4.1 Is there a relationship between climate change and past forest loss?

Our analyses show that there are significant correlations between both temporal trends and velocities of climate variables with increased forest loss in the study area. Despite warming up to 0.051°C year^−1^ on average, and the known detrimental effects of warmer temperatures and drought on forest growth (Bonan, 2008; Chaturvedi et al., 2011; Gopalakrishnan et al., 2011), we found that temperature decreases and slower warming were unexpectedly the strongest predictors for forest loss. Though the mechanism behind this relationship is unknown, as deforestation and encroachment have been prevalent the area for many years, much of the forest exists at higher elevations where temperatures tend to be cooler. The trend is also likely affected by high forest loss in the NE and CNE where there is an anomalous cooling patch, thought to be caused by a growing aerosol haze (Ross et al., 2018). Many studies contrastingly predict temperature increases in these regions and expect forests to be adept at coping with warming (Chaturvedi et al., 2011; Ravindranath et al., 2005), our research suggests a re‐evaluation of the climate threats to forests in this region given the substantial cooling. Although cooler temperatures in the tropics are not thought to be a direct threat to forests, there is the potential for indirect effects caused by additional pressure on people in the region, for example, reducing agricultural yields or inducing additional fuelwood collection.

Relationships between precipitation and forest loss were also common in both the study area‐scale and regional models, though the trends were highly variable both regionally and seasonally. Precipitation decreases and faster velocities were most associated with increased losses. This trend was strongest in the NE, EHR and Northern Western Ghats, but did not appear as a correlate of forest loss in the respective regional models as drying spots occurred on the borders between regions. Precipitation increases were also associated with increased loss in some regions. This has been found in other studies (Maringer et al., 2021; Neumann et al., 2017) and although appearing counterintuitive can arise due to increased competition after forest growth spurts (Condit et al., 2004; McDowell, 2018) and the decoupling between precipitation and soil moisture which, in areas of groundwater depletion like NW India, is common (Condon & Maxwell, 2019; Zaveri et al., 2016).

Mapping of trends in areas of high forest loss (<20 km^2^) across the study area revealed that most experienced reductions in precipitation, particularly during the post‐monsoon and winter seasons, and year‐round in the NE and EHR regions. This is concerning for future forest persistence, with adequate precipitation and soil moisture often critical for forest growth (Seidl et al., 2017). However, these trends may not have been captured in the regional models as the high forest loss areas often overlapped more than one monsoon region and thus the trend may be separated into two regional models. This should be taken into account in future studies, with models used on contiguous areas of high forest loss to determine the role of climate change to specific areas of high forest loss. Additionally, many areas experienced warming winter temperatures coupled with reduced precipitation. Of particular concern are the Western Ghats and the Hilly region, both areas of conservation importance and high endemism. These results support previous studies detailing forests in these regions to be a high risk of climate change effects in the future (Chaturvedi et al., 2011; Ravindranath et al., 2005; Sharma et al., 2015; Upgupta et al., 2015). Importantly, though warming and drying conditions were most common in areas of high forest loss, these are not the only indicators of a climate change impact and substantial cooler, and wetter conditions were prevalent in the high forest loss area located at the nexus of the CNE, WC and PEN regions.

### 4.2 Are there seasonal and regional variations in the climate–forest loss relationship in the study area?

Regionally, the way climate affected forest loss varied greatly, both in the amount of exposure to different variables and in the effect directions of relationships. Forests in some regions, such as the NW, had a greater variety of climate variables correlated with forest loss. Here, high seasonal variation in the climate variables associated with forest loss could require different strategies for conservation throughout the year to tackle potential winter droughts and summer flooding. With some of the lowest amounts of forest cover in the study area due to its aridity, even small losses have large implications for the overall forest cover. These analyses support predictions of the high vulnerability of remaining NW forests to climate changes (Chaturvedi et al., 2011; Das & Behera, 2019; Gopalakrishnan et al., 2011). The NE, where loss is highest, was only associated with one climate variable. This region is thought to be largely resilient to projected climate changes due to lower exposure, and more resilient forest types (Chaturvedi et al., 2011; Gopalakrishnan et al., 2011). Known for its high levels of shifting cultivation and agricultural encroachment (Lele & Joshi, 2009; Lele et al., 2008), land use change and other factors likely still play a main role in forest loss here.

Every season appeared as a correlate of forest loss in the models and there was no clear dominant season that affected forest loss. The diversity in seasonal contributions to forest loss between regions highlights the diversity found in climate and forest type across the study area and illustrates the array of challenges forests could face if seasons show diverging trends.

Precipitation trends fluctuated more than temperature throughout the year, varying greatly by season, which species may find harder to adapt to than a unidirectional climate change. Interestingly, the fastest velocities and largest changes in precipitation occurred in different seasons (pre‐monsoon and monsoon) to those of temperature (post‐monsoon and winter). Though this result could provide seasonal respite from overlapping high velocities, it could mean that forests are exposed to potential year‐round climate stress. In addition, our analyses revealed several occurrences where adjacent seasons had diverging trends. This may have repercussions for processes of growth and reproduction, as existing evidence shows that seasonal climate patterns can impact plant phenology in subsequent seasons (Chen et al., 2017; Cook et al., 2012; Harvey et al., 2019; Laube et al., 2013).

For management strategies to be effective, they will need to be able to evolve with the seasons, be regionally specific and account for difficult transition periods. The variation found in this study provides evidence for a need for a diverse range of strategies not only throughout the study area but also throughout the year.

### 4.3 Are forests exposed to high and overlapping climate velocities and is forest loss greater in these areas?

Forests were exposed to high velocities of both climate variables, and faster velocities were found to be correlated with areas of higher forest losses in the models. However, faster velocities did not always denote more forest loss. A key example of this is the relationship between negative monsoon temperature velocities and increased loss. With no occurrences of declining monsoon temperatures, only lower increases, velocities must be indicative of slower increases in temperature. As such, it is likely that high velocities are not sole determinants of forest loss. Although the relationship between higher velocities and forest loss is not always detrimental, it is promising that no high forest loss areas had year‐round exposure to high velocities. Further research is needed to understand when high velocities become detrimental to forests.

Encouragingly, overlaps of fast climate velocities of temperature and precipitation were uncommon and generally covered small areas. This supports other studies that have shown spatial heterogeneity in temperature and precipitation velocities (Garcia et al., 2014; Heikkinen et al., 2020). The exception in the Northern Western Ghats could be concerning due to increased drought and fire risk in an area that covers seven protected areas within a biodiversity hotspot (Figure S8), and is already threatened by encroachment by agriculture and extensive fragmentation (Jha et al., 2000; Sharma et al., 2015). In addition, all three of the forest loss hotspots identified by this study received singular high velocities at some point during the year, with the Northeast and eastern Hilly regions experiencing some of the fastest negative precipitation velocities in every season. This prolonged exposure to rapid changes in climate could mean that species here are under additional pressure to move or adapt to climate sooner. The Northeast and eastern Hilly regions host some of the most biodiverse forests in the study area (Chatterjee et al., 2006; Lele & Joshi, 2009) and fast velocities of changing climate here add stress to species already experiencing high levels of threat from land use change (Lele & Joshi, 2009; Ramakrishnan, 2007).

Precipitation velocities generally ~5–10 km year^−1^, were much larger than those recorded for temperature, which were 0.6 km year^−1^ at their fastest. These precipitation velocities are likely unattainably fast even for far more mobile species than trees, which under ideal conditions are expected to move a kilometre a year at best (Corlett & Westcott, 2013). The velocities recorded for precipitation in the study area (annual mean at 3.98 km year^−1^) are high compared to other studies including the global mean of 0.22 km year^−1^ (Kosanic et al., 2019; Loarie et al., 2009; Van der Wal, 2013). However, velocities of temperature (annual mean at 0.029 km year^−1^) are much lower than the global average of 0.42 km year^−1^ (Loarie et al., 2009; Van der Wal, 2013). Our results suggest that precipitation velocities may be greater in the tropics than those in temperate regions but the same may not be true for temperature. For species capable of tracking climate, precipitation velocities could be a great concern as the speeds in which species would need to travel to reach their preferred climate may be too quick to traverse.

### 4.4 Can climate velocity provide additional understanding of a forests’ risk to climate change?

This study found the metric of climate velocity to provide additional information compared to traditional temporal trend analysis as it provides a measure of, and a suggested repercussion of, the spatial variability in the climate variable of interest. Different relationships between climate change and forest loss were found in the study area due to the effect of the spatial gradient and, if forests respond in the way that the velocity mechanism expects, climate velocity should be an important component of management plan for protecting forests in India, Jammu, Kashmir and Ladakh. The metric has been used in the past to assess the vulnerability of areas to future climate change and the utility of protected areas in the future (Arafeh‐Dalmau et al., 2021; Fuentes‐Castillo et al., 2020). Areas where climate velocity is low are likely to be key refuges for many species in the future and management strategies should take this into account and ensure these low velocity areas are as protected from multiple threats as possible. Additionally, climate velocity can identify areas that are climatically heterogeneous and are key refuge areas for species. Ensuring that there are corridors between high velocity, spatially homogeneous areas, and low velocity, heterogeneous refuges could help many species transition between climatically unsuitable or rapidly changing areas to more suitable, refuge sites as well as ensuring protected areas are large enough to provide a variety of climate conditions for species (Brito‐Morales et al., 2018). The majority of the protected areas in the study area do not fall within the high velocity areas for either precipitation or temperature (Figure 5 and Figure S8). This is promising as they lie in potential refuge areas for species and the protected area status may relieve pressures from other stressors such as land use change. Many of the areas with a higher coverage of protected areas, such as the Western Ghats, are also in mountainous, and therefore climatically heterogeneous landscapes, offering more protection (Brito‐Morales et al., 2018; Loarie et al., 2009). However, it is concerning that there appears to be few protected areas in locations of high climate velocities, such as the central areas of the PEN region and the PEN, WC and CNE nexus. The lack of protected areas across these more exposed locations could mean that there are not the ecological corridors available for species to adjust their distribution safely with climate change. The majority of the study region lies within India, and the country's National Biodiversity Target 6 aimed to have 20% of the country's land area covered by protected areas by 2020 (Convention on Biological Diversity, 2020). According to the ENVIS reports (ENVIS, 2020), India fell short of this target in 2020 reaching just 5% coverage in protected areas (including areas protected under lower protection status such as Wildlife Reserves). The results from our study could help to inform placement of new protected areas in the country to reach the 20% target with climate change trajectories in mind.

We find climate velocity to be a valuable metric, especially when used at a large scale where it can identify areas where the speed of climate change could be a concern for species persistence. However, this metric is known to lack biological realism at present and there are several caveats to its efficacy in indicating species vulnerability to climate change (Brito‐Morales et al., 2018; Carroll et al., 2015; Hamann et al., 2015). In particular, we note concerns around comparing temperature and precipitation velocities. Absolute values of precipitation will usually be much higher than temperature but their values are not comparable in terms of effect on species. Additionally, the fastest velocity in this study, −97 km year^−1^, was located on a mountain plateau, a small area of low spatial gradients but surrounded by a myriad of valleys (potential climate refuges). We stress that a key area of future study should be assessing the biologically realism of the spatial gradient aspect of climate velocity metrics specifically for forests before using this metric to obtain realistic estimates of forest species risk. We also stress that this metric should be integrated with more biologically realistic parameters if used in future modelling studies.

Despite these caveats, the metric has provided additional information on the general climate risk of a region not possible from conventional temporal trend data. It highlights areas of continuous homogeneous climate which may have reduced opportunities for species to find climate refuges, particularly evident for temperature in the study area where the spatial gradient was considerably lower. This can be useful in planning areas for long‐term conservation (Heikkinen et al., 2020; Loarie et al., 2009). It is also meaningful when considering the breadth of species reliant and relied on by tropical forests that are capable of moving to more climatically suitable, available areas.

### 4.5 Methodological considerations and future directions

This study provides novel insight into the potential climate variables leading to forest loss in a tropical‐subtropical system with a uniquely large, but policy‐relevant, scale. However, there are associated limitations that are highlighted below to enable improvements in future studies.

#### 4.5.1 The use of population density as a land use proxy

Previous studies have shown human pressures, such as increasing land use changes, as a major causes for forest declines in the study area (Gupta, 2007; Meiyappan et al., 2017; Padalia et al., 2019; Sudhakar Reddy et al., 2016). Higher population densities were expected to increase pressure on forest resources leading to more loss. However, our results, using the proxy of population density, do not support this. Although higher population densities are likely to put additional pressure on forest resources, many densely populated areas have little forest cover left resulting in loss occurring further from the source of the demand, geographically uncoupling the relationship between population density and demand on forest resources. Forest encroachment has also been linked to other socio‐economic drivers such as out‐migration of labourers and infrastructure such as irrigation facilities (Meiyappan et al., 2017). As population density does not account for these factors and showed a relatively small effect on forest loss in the models, the contribution of other human pressures, for example, land use change and infrastructure, to forest loss trends remains an open question. Future studies will aim to investigate the relative contributions of both human pressures and climate change in conjunction to forest loss.

#### 4.5.2 The importance of spatial and temporal scales

These analyses find that trends are misleading when focusing solely on annual climate averages. This is particularly the case for precipitation, where seasonal variation is masked in annual averages by strong opposing monsoonal trends. Focusing solely on annual averages in this study results in concluding no effect of climate on forest loss. This has repercussions such as underestimating future projected losses, dismissing interactions with other stressors and missed opportunities for protection. We stress that in areas with high seasonality, using seasonal data is necessary at the very least.

We also highlight the importance of utilizing an appropriate spatial scale in large‐scale analyses. The results obtained for study area scale models and regional models differed greatly in this study. The use of regional models highlighted large variation in climate drivers of loss across the study area, but also separated climatic trends and contiguous areas of forest. This is of particular concern in the border districts of the CNE, WC and PEN regions which contained contiguous areas of high forest loss and homogeneous climatic trends, but which were segregated in the regional models, potentially lessening the impact of climate trends observed.

Consequently, policy and conservation practice decisions need to take into account the different trends observed at different spatial and temporal scales as these could infer different management solutions. For example, assessing the effects of climate change on forests using data at the study area scale and using annual averages may lead conservation managers and policymakers to conclude that climate change is having minimal effect on forests. However, this overlooks the significant seasonal effects causing stress on forests, as well as the vast regional differences in climate threats. Conservation strategies and policies will be most effective where they take into account the trends across multiple spatial and temporal scales.

#### 4.5.3 Lag times and contribution of plantation forests

Forests often have lagged responses to changes in climate (Bertrand et al., 2011; Tei & Sugimoto, 2018). However, these can be highly variable between species and there is no clear consensus on the length of such lags (Bertrand et al., 2011; Corlett & Westcott, 2013; Kosanic et al., 2019; Liang et al., 2018). Therefore, it was difficult to account for without detailed context‐specific information at the species level and as such lags were not considered in this study. The forest data used in this study also do not discriminate between natural and plantation forests, a known concern with other forest data in the study area such as the Forest Survey of India datasets (Puyravaud et al., 2010; Sudhaker Reddy et al. 2016). Some losses recorded in this study are possibly due to harvesting of tree plantations and not natural forests. Future studies would benefit greatly from the creation of forest cover maps that can distinguish between natural and plantation forests (Puyravaud et al., 2010). Furthermore, this dataset only determines forest losses as a result of climate change and not forest degradation or changes in floristic composition. Further study is needed to assess the effects of climate change on other components of forest change.

## 5 CONCLUSIONS

We show, for the first time, that climate change has played a role in past forest loss in India, Jammu, Kashmir and Ladakh and provide the first characterization of climate velocity in the area. We highlight a concern for future forest loss due to emerging drying trends and the locations and magnitude of singular high velocities in area's remaining forest strongholds. This study highlights the issues around spatial and temporal scales leading to misrepresentation of climatic contributions to forest losses, particularly in ecologically and climatically diverse systems. Although this study shows climate to contribute to forest loss in the study area it also supports that other stressors, particularly land use change, likely still play a major role. Currently, India, where the majority of our study area resides, is in the process of creating a new National Forest Policy to replace the last, which was written in 1988. Therefore, this is a crucial time for policies to be considering the multiple threats that forests in the country face. Adaptable management strategies built from up‐to‐date research is even more important as the area's forests face unprecedented threats on multiple fronts. As climate changes become more extreme, an understanding of how stressors interact will be of paramount importance in preserving forests and biodiversity within India, Jammu, Kashmir, and Ladakh. In light of this, future studies should aim to quantify different aspects of the climate–forest relationship in the region, particularly the response of different tree species to climate, prevalence of extreme events for example, drought, interactions between climate and other stressors, the lag time, and the effects of climate‐related forest loss on other aspects of biodiversity. Studies, such as this, where other drivers of forest loss are explored, can help to inform conservation policy and practice on national and regional levels, leading to more successful and cost‐effective management programmes, especially as climate changes become more prevalent.

## Supporting information

Supplementary Material

## Data Availability

The data that support the findings of this study are openly available in the University of Reading Research Data Archive at https://doi.org/10.17864/1947.000364.
