# Supplementary material for: The role of climate in past forest loss in an ecologically important region of South Asia
Source: Glob Chang Biol. 2022 Mar 23;28(12):3883–901. doi: 10.1111/gcb.16161 (PMC9314953; doi:10.1111/gcb.16161)
Supplement: Supplementary file 1 — Supplementary Material [file GCB-28-3883-s001.docx]

**Supplementary Material**

**Table S1|** List of excluded districts due to having less than 0.1km^2^ of forest cover at the start of the study period

| District | State | District Area (km^2^) | Forest cover (km^2^) | Forest loss (km^2^) | Percent of total forest cover lost (%) |
| --- | --- | --- | --- | --- | --- |
| Churu | Rajasthan | 17075.11 | 0 | 0 | 0 |
| Jaisalmer | Rajasthan | 38637.60 | 0 | 0.0020 | 0 |
| Bikaner | Rajasthan | 26965.39 | 0 | 0.0027 | 0 |
| Jodhpur | Rajasthan | 22842.40 | 4.49E-04 | 0.0158 | 1.00E+02 |
| Patan | Gujarat | 6026.34 | 0.0016 | 0.0113 | 100 |
| Barmer | Rajasthan | 28372.88 | 0.0028 | 0.0069 | 100 |
| Hanumangarh | Rajasthan | 8912.64 | 0.0066 | 0.0174 | 100 |
| Nagaur | Rajasthan | 17676.90 | 0.0123 | 0.0158 | 100 |
| Yanam | Puducherry | 31.65 | 0.0436 | 0 | 0 |
| Sirsa | Haryana | 4236.29 | 0.0721 | 0.0221 | 30.67 |
| Hyderabad | Telangana | 178.60 | 0.0725 | 0.0242 | 33.39 |
| Bhilwara | Rajasthan | 10469.55 | 0.0764 | 0.0390 | 51.03 |
| Ganganagar | Rajasthan | 11679.58 | 0.0856 | 0.1044 | 100 |

**
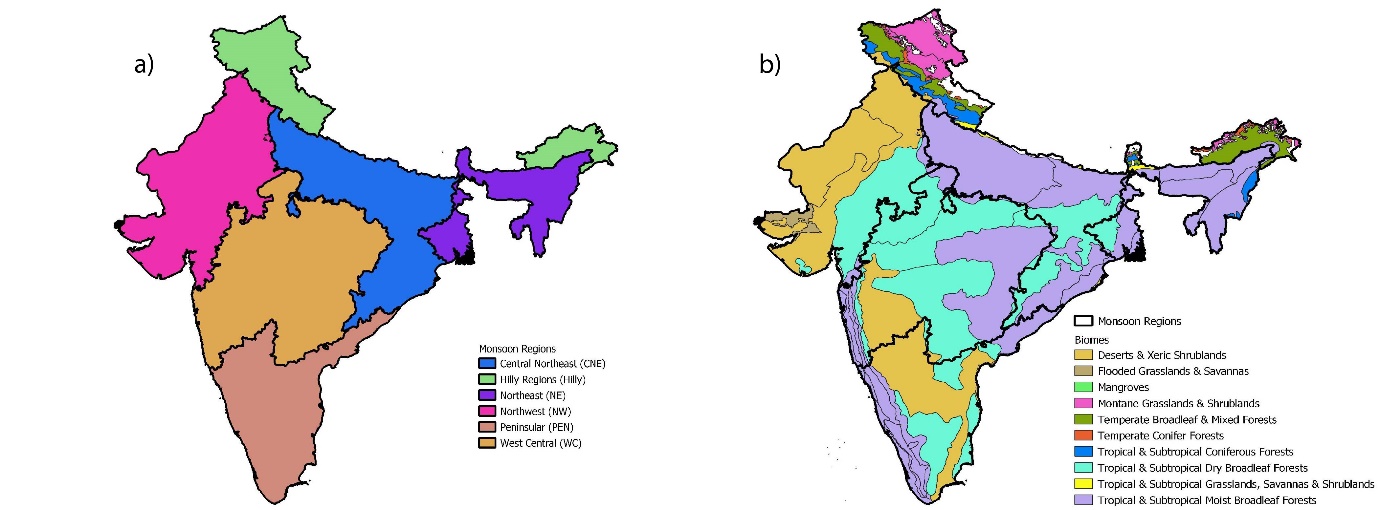
**

**Figure S1| a):** The six monsoon regions used in this study; Northeast (NE), Northwest (NW), Central Northeast (CNE), West Central (WC), Peninsular (PEN) & Hilly. The map was created to display the Homogenous Monsoon Regions of India outlined by the Indian Institute of Tropical Meteorology. **b):** The ecological biomes of India. Most of India is located in the biomes of Tropical and Subtropical Moist Broadleaf forests, Dry Broadleaf forests and Deserts and Xeric Shrublands. The map is a clipped version of the global extent created by Dinerstein et al., (2017)

**
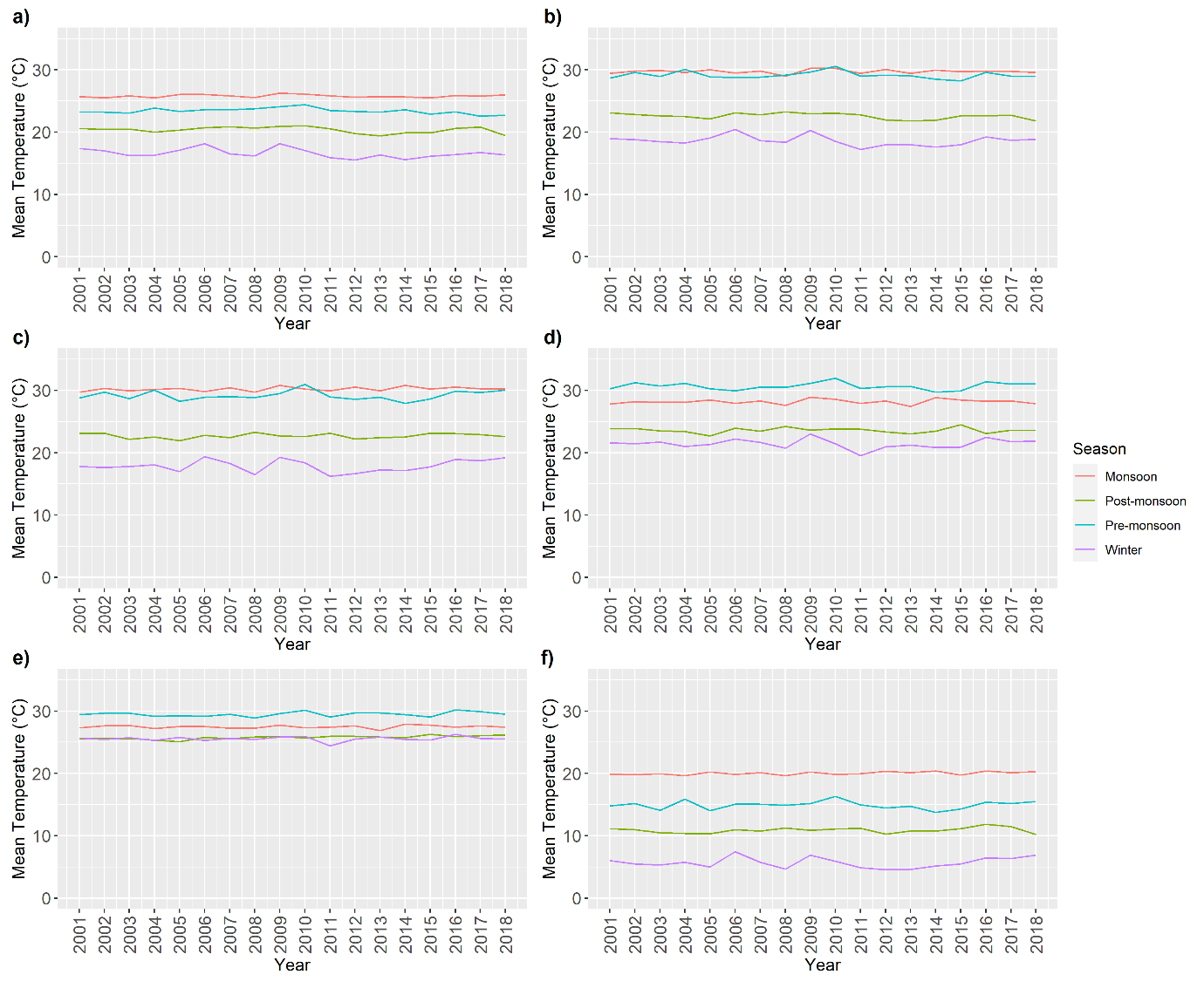
**

**Figure S2|**Trends in seasonal mean temperature (⁰C) across all six monsoon regions, a) Northeast, b) Central Northeast, c) Northwest, d) West Central, e) Peninsular, f) Hilly, for the time period 2001-2018. Different seasons are depicted as individual lines; monsoon (red), post-monsoon (green), pre-monsoon (blue) and winter (purple).


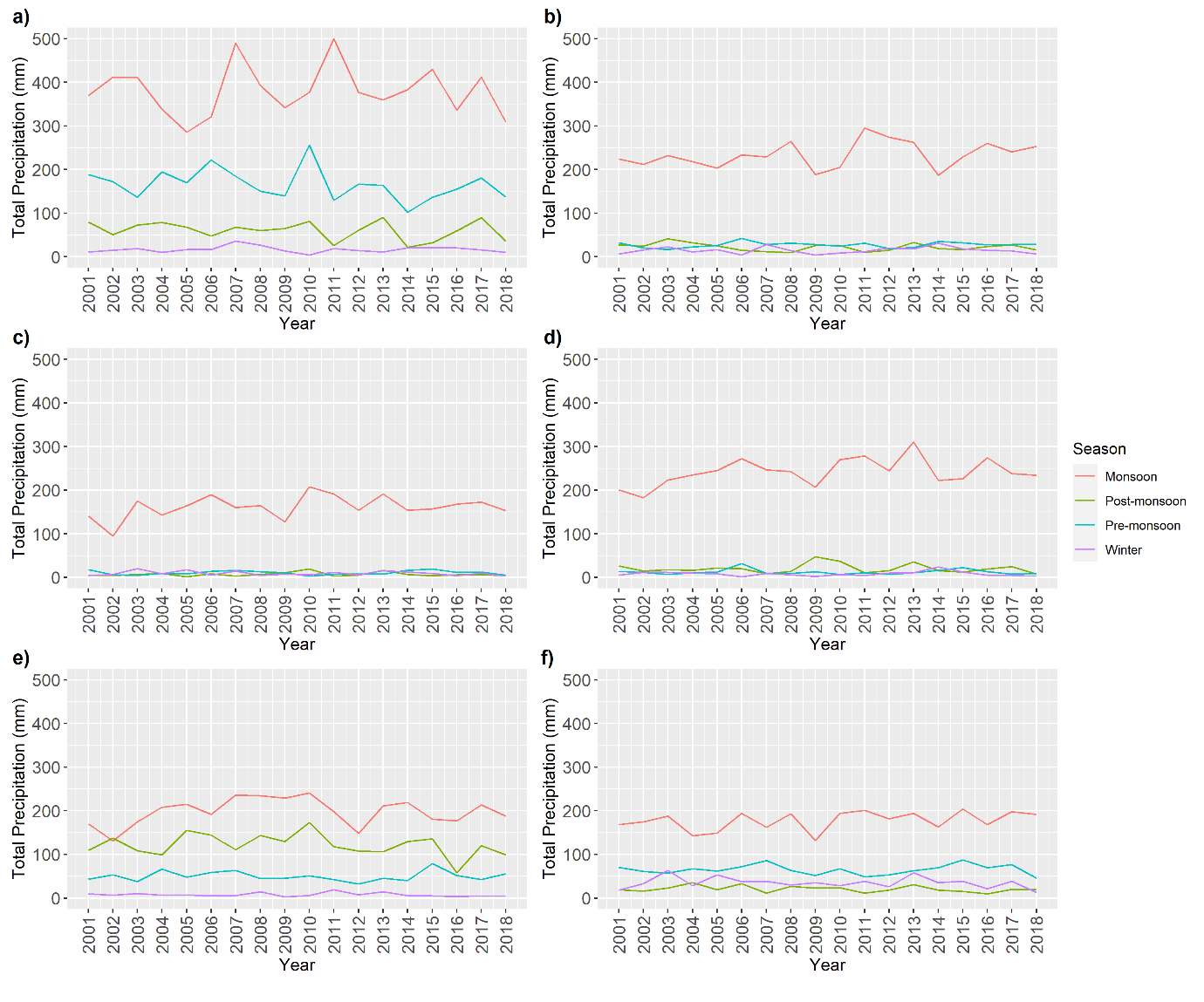


**Figure S3|**Trends in seasonal total precipitation (mm) across all six monsoon regions, a) Northeast, b) Central Northeast, c) Northwest, d) West Central, e) Peninsular, f) Hilly, for the time period 2001-2018. Different seasons are depicted as individual lines; monsoon (red), post-monsoon (green), pre-monsoon (blue) and winter (purple)

**
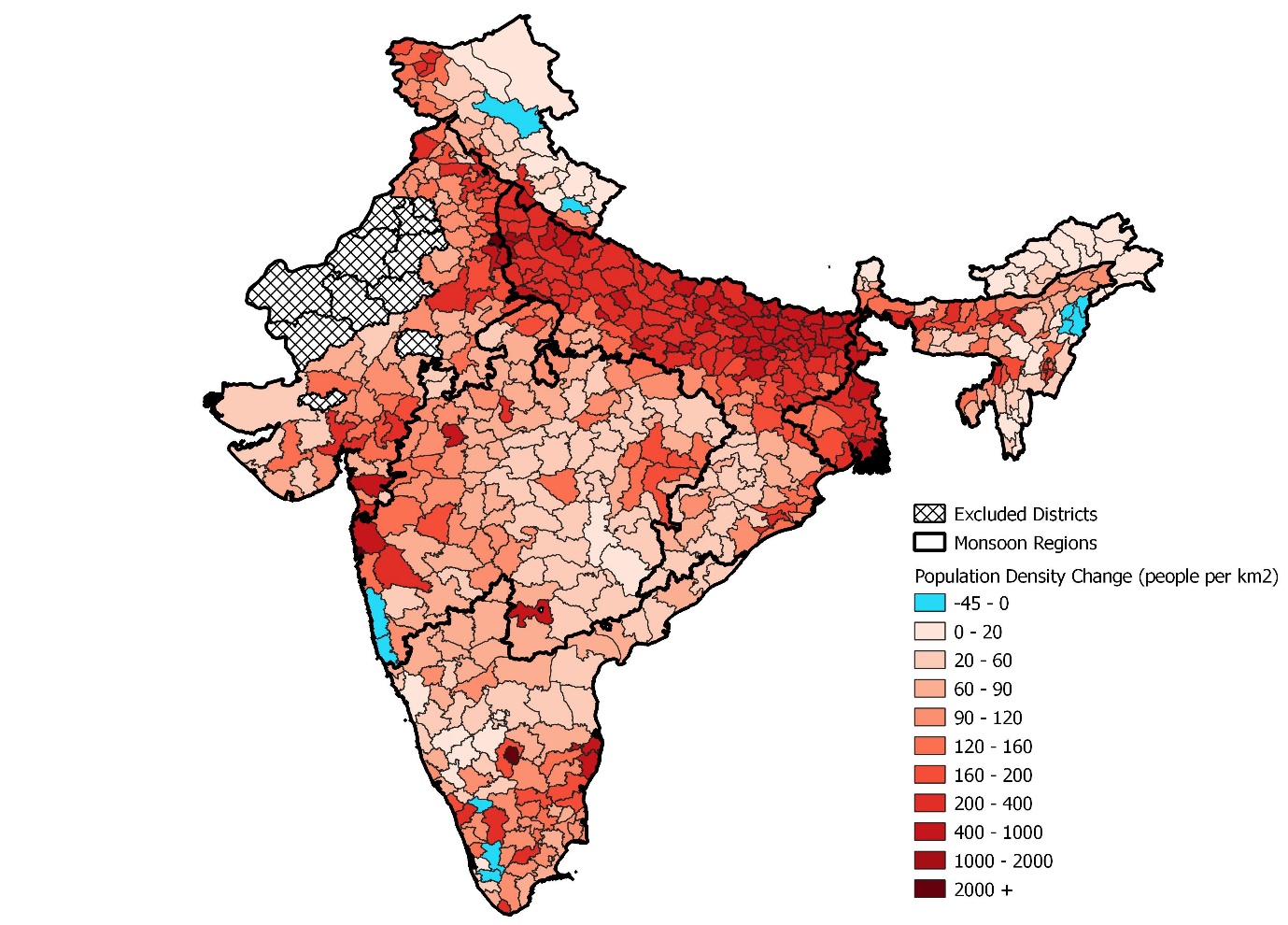
**

**Figure S4 |** Population density change (people per km^2^) between the years 2000-2020 in the districts of India. The thick black lines show the borders of the monsoon regions.


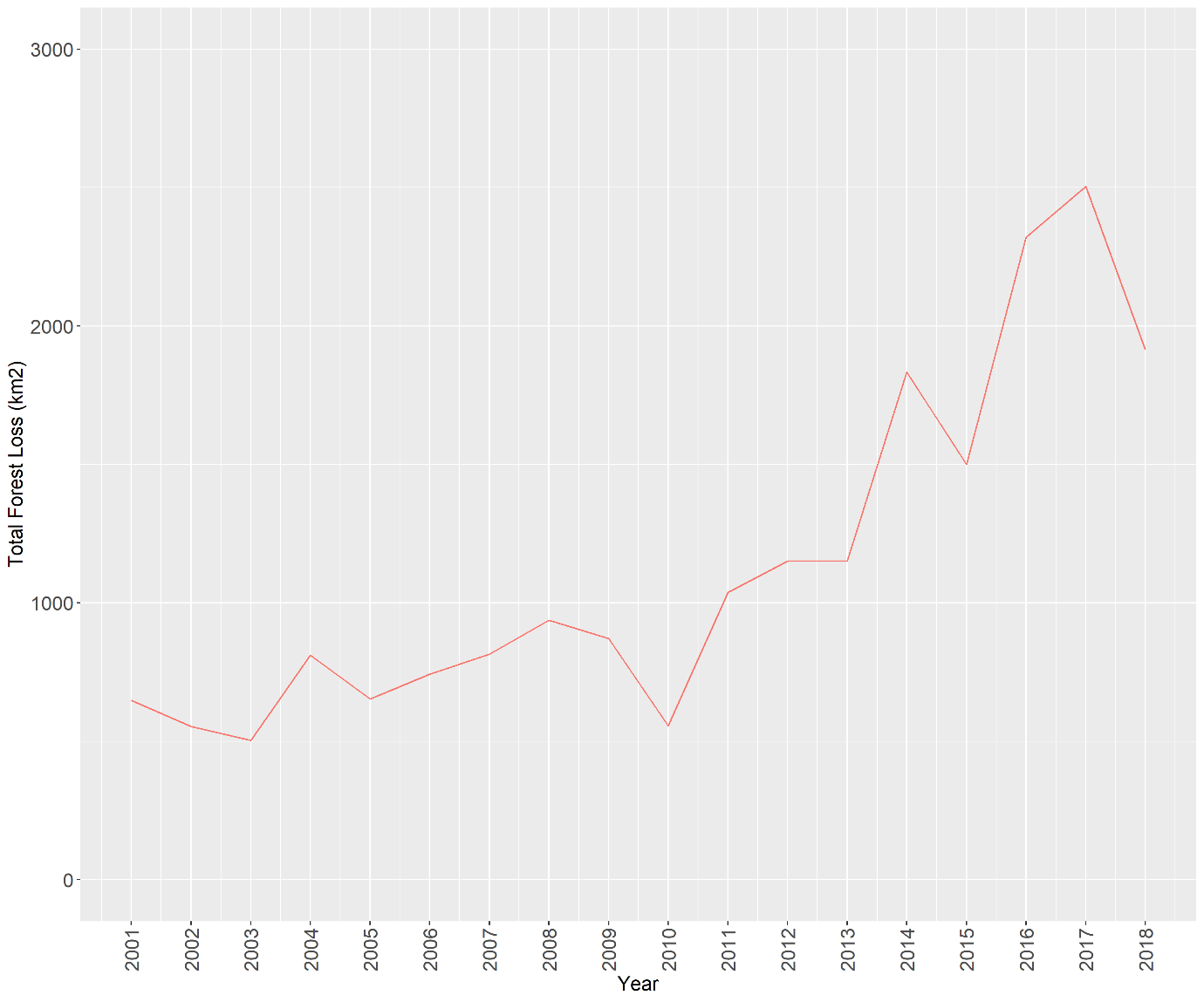


**Figure S5|** The increase in total annual forest loss on a national level between the years 2001-2018 in km^2^


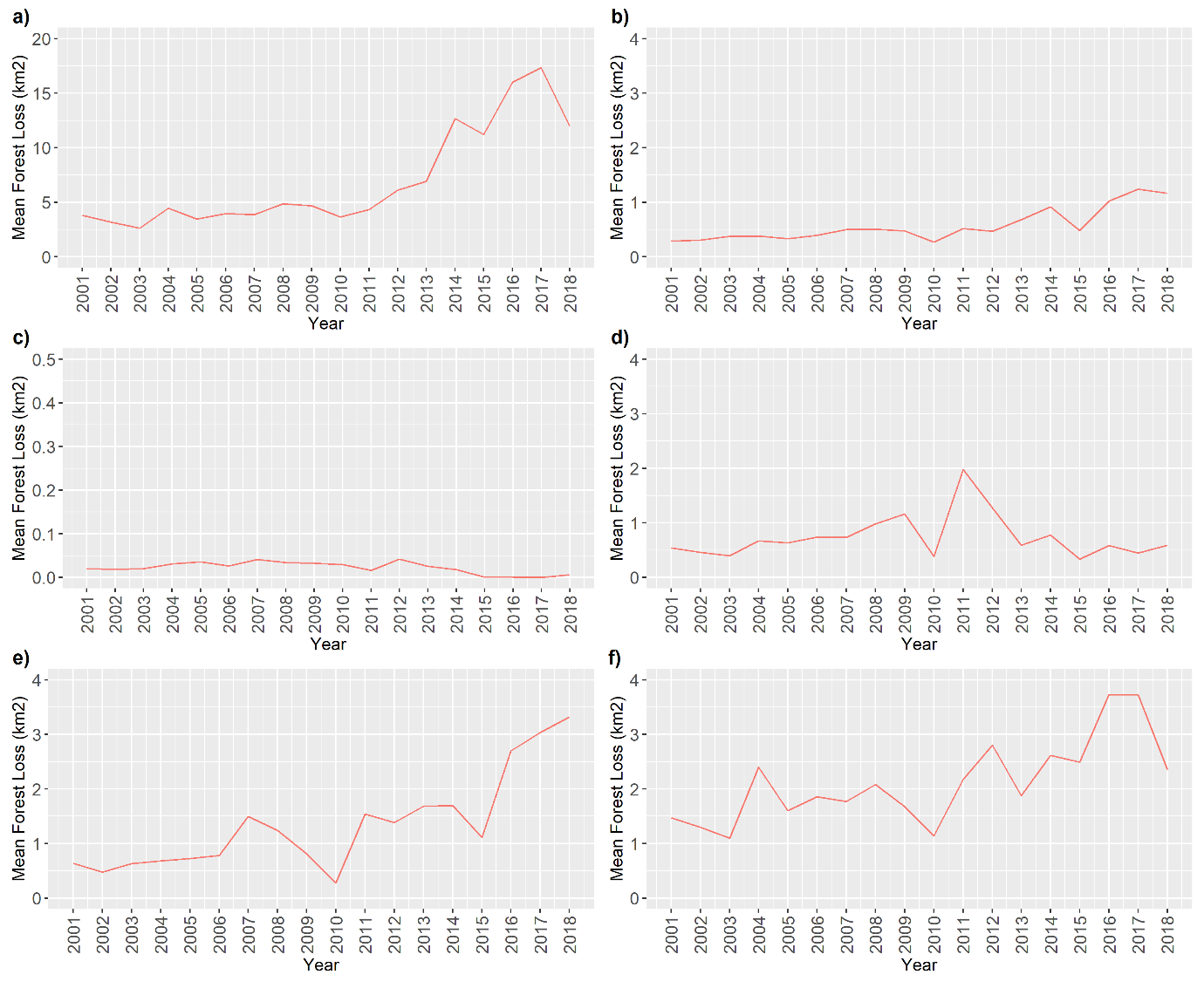


**Figure S6|** The mean annual forest loss per district per year in the monsoon regions of India, a) Northeast, b) Central Northeast, c) Northwest, d) West Central, e) Peninsular, f) Hilly, between the years 2001-2018. Note different axis extents have been used to ensure trends are visualised.

**
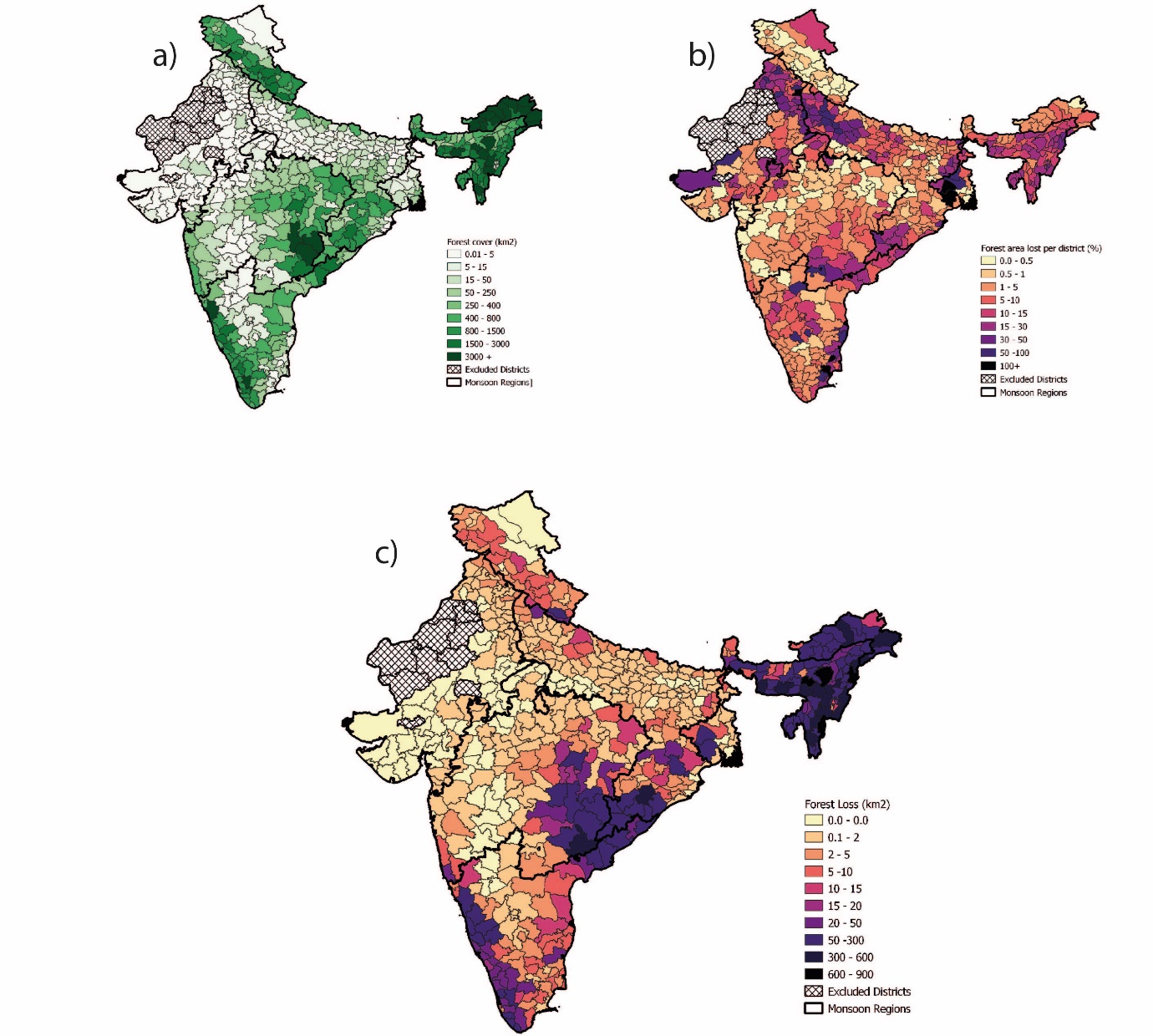
**

**Figure S7| a)**: The forest cover in km^2^ of each district in India in the year 2000, **b)**: The percent of each district’s forest cover that was lost between the years 2001-2018, **c)**: The total forest lost in each district between the years 2001-2018 in km^2^. Much of the country’s forest cover is located in the Northeast and along the east and southwestern coasts. The highest percentage of forest cover lost are spread across the country with hotspots in the Northwest and Northeast. Total forest loss is greatest in the Northeast, central west coast and southwestern areas, where forest cover is also high.

**
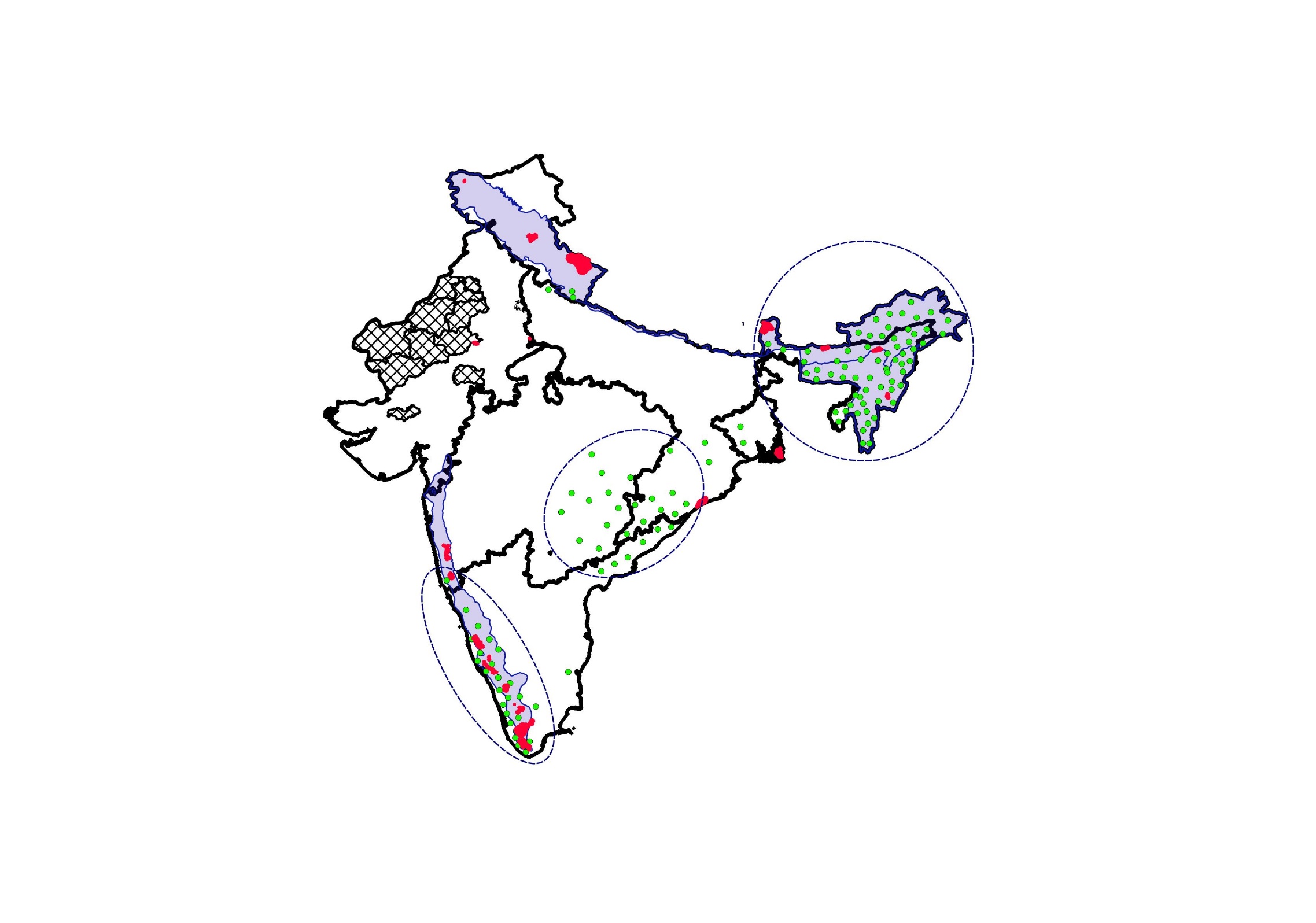
**

**Figure S8|** Districts with forest losses >20km^2^ during the time period 2001-2018. Green dots indicate the central point of a district that had a forest loss area greater than 20km^2^ during the time period 2001-2018. The three areas of high forest loss are highlighted with blue circles. Protected areas are marked in red (UNEP-WCMC & IUCN, 2021). Biodiversity hotspots are marked as blue filled in areas (Hoffman, et al., 2016). The map is split up into the homogenous monsoon regions and hashed districts display those excluded from the study.

###

**
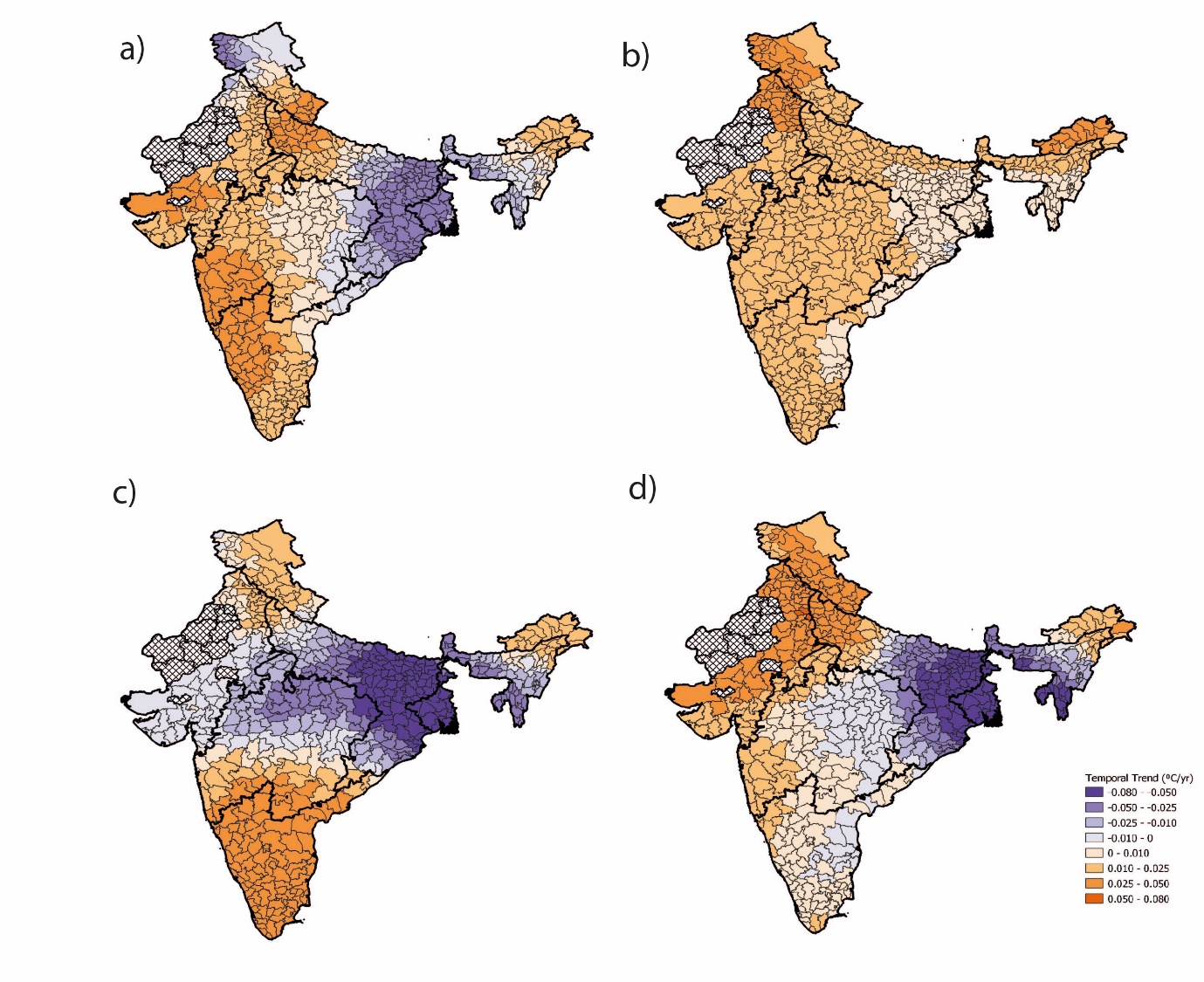
**

**Figure S9|** Seasonal temperature temporal trends in ⁰C/year of each district for the time period 2001-2018. In a clockwise direction the seasons depicted are as follows; pre-monsoon, monsoon, post-monsoon and winter. The black outlines show the borders of the monsoon regions.

**
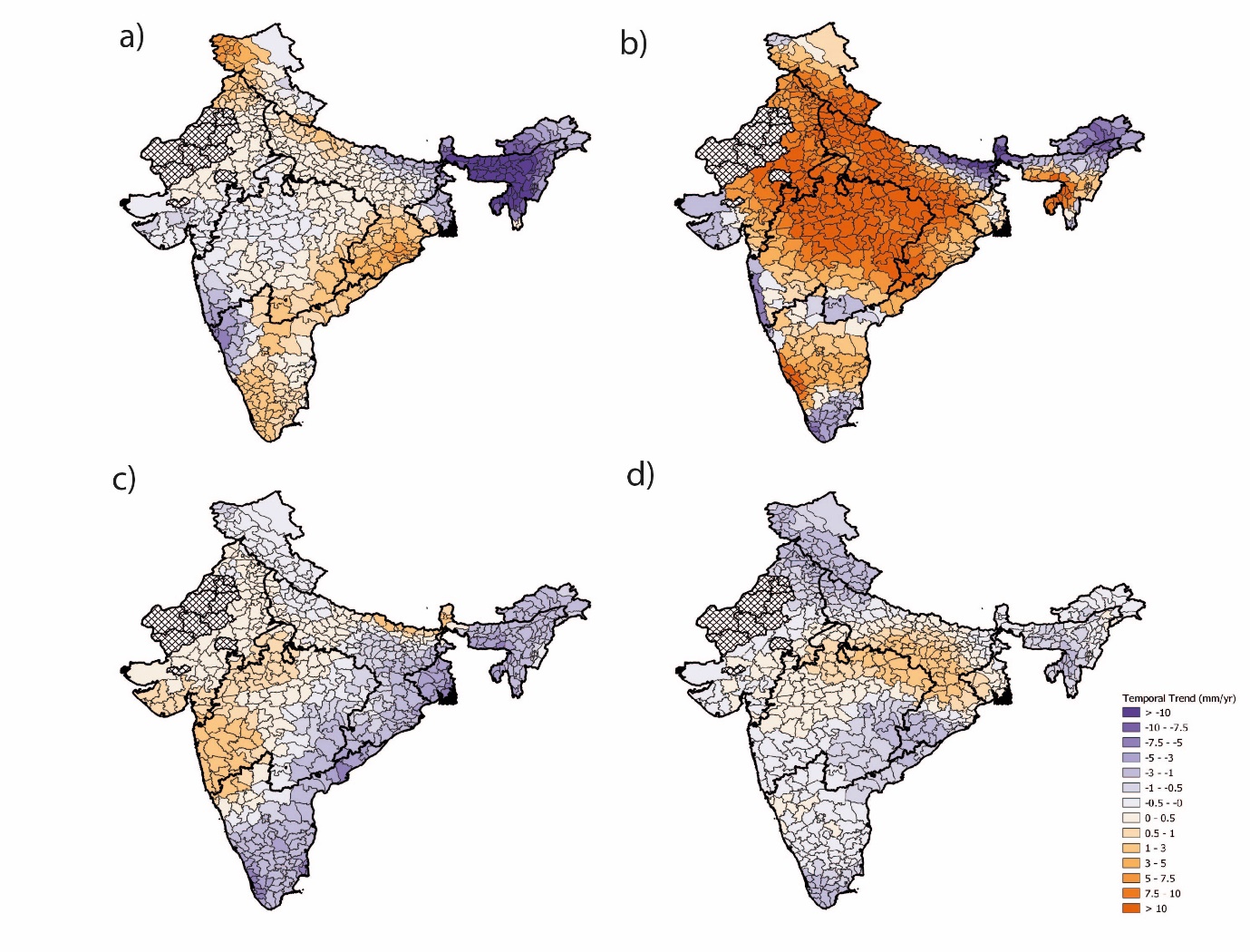
**

**Figure S10|** Seasonal precipitation temporal trends in mm/year of each district for the time period 2001-2018. In a clockwise direction the seasons depicted are as follows; pre-monsoon, monsoon, post-monsoon and winter. The black outlines show the borders of the monsoon regions

**Table S2|** The range and average values of precipitation and temperature velocities of annual and seasonal variables.

| Season | Range of Precipitation Velocities | Mean | Range | Range of Temperature Velocities | Mean | Range |
| --- | --- | --- | --- | --- | --- | --- |
| Annual | -13.752 - 34.320 | 3.981 | 48.072 | -0.321 - 0.298 | 0.029 | 0.619 |
| Monsoon | -10.152 - 41.388 | 4.881 | 51.54 | -0.010 - 0.272 | 0.118 | 0.282 |
| Post-  monsoon | -14.550 - 11.254 | -0.903 | 25.804 | -0.725 - 0.440 | -0.090 | 1.165 |
| Pre-  monsoon | -97.586 - 13.086 | -0.008 | 110.672 | -0.334 - 0.403 | 0.042 | 0.737 |
| Winter | -16.458 - 19.034 | -0.589 | 35.492 | -0.606 - 0.489 | -0.025 | 1.095 |

**Table S3|** The range and mean values of precipitation and temperature spatial gradients between seasons

| Season | Temperature Range (Mean) | Precipitation Range (Mean) |
| --- | --- | --- |
| Pre-Monsoon | 0.1-0.38 (0.128) | 0.1-4.63 (0.549) |
| Monsoon | 0.1-0.33 (0.125) | 0.1-19.40 (2.271) |
| Post-Monsoon | 0.1-0.36 (0.126) | 0.1-5.13 (0.383) |
| Winter | 0.1-0.36 (0.127) | 0.1-2.30 (0.203) |

**Reference**

Dinerstein, E., Olson, D., Joshi, A., Vynne, C., Burgess, N. D., Wikramanayake, E., Hahn, N., Palminteri, S., Hedao, P., Noss, R., Hansen, M., Locke, H., Ellis, E. C., Jones, B., Barber, C. V., Hayes, R., Kormos, C., Martin, V., Crist, E., … Saleem, M. (2017). An ecoregion‐based approach to protecting half the terrestrial realm. BioScience, 67(6), 534–545. <https://doi.org/10.1093/biosci/bix014>

Hoffman, M., Koenig, K., Bunting, G., Costanza, J., & Williams, K. J. (2016). Biodiversity hotspots (version 2016.1). Zenodo. <https://doi.org/10.5281/zenodo.3261807>

UNEP‐WCMC and IUCN. (2021). Protected planet: The world database on protected areas (WDPA) [Online], [January 2021]. UNEP‐WCMC and IUCN. Retrieved from www.protectedplanet.net
